# Supplementary material for: MAP kinase ERK5 modulates cancer cell sensitivity to extrinsic apoptosis induced by death-receptor agonists
Source: Cell Death Dis. 2023 Nov 2;14(11):715. doi: 10.1038/s41419-023-06229-6 (PMC10622508; doi:10.1038/s41419-023-06229-6)
Supplement: Supplementary file 5 — Supplementary figures [file 41419_2023_6229_MOESM5_ESM.pdf]

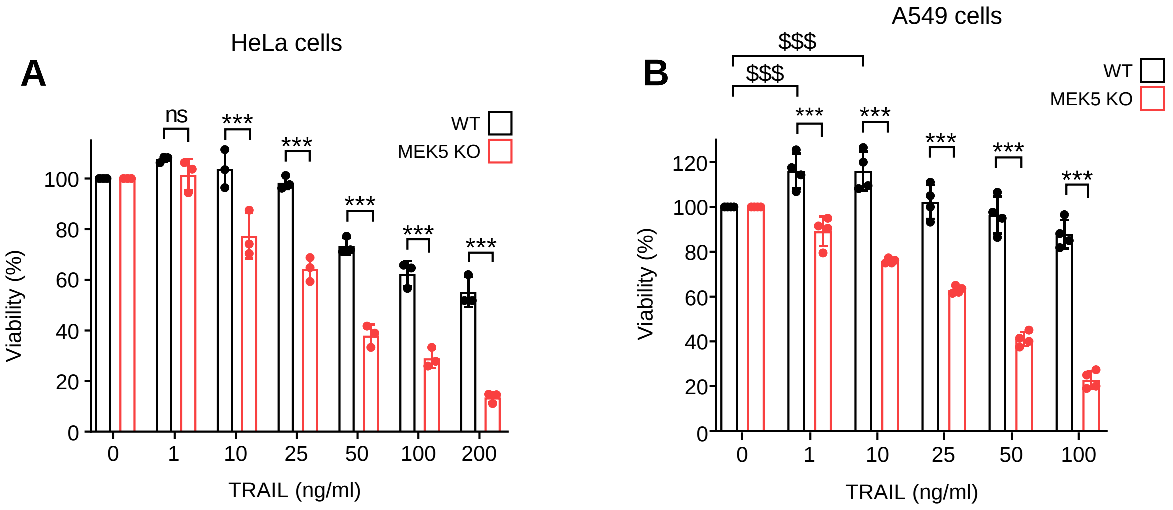

Supplementary Figure 1. MEK5 genetic deletion confers increased sensitivity to TRAIL-induced toxicity in cervical cancer Hela and NSCLC A549 cells. A. Cervical carcinoma (HeLa) or **B.** NSCLC cells (A549) were treated with TRAIL for 24 h and cell viability was analyzed. ***p < 0.001 (one-way ANOVA followed by Bonferroni multiple comparison test).

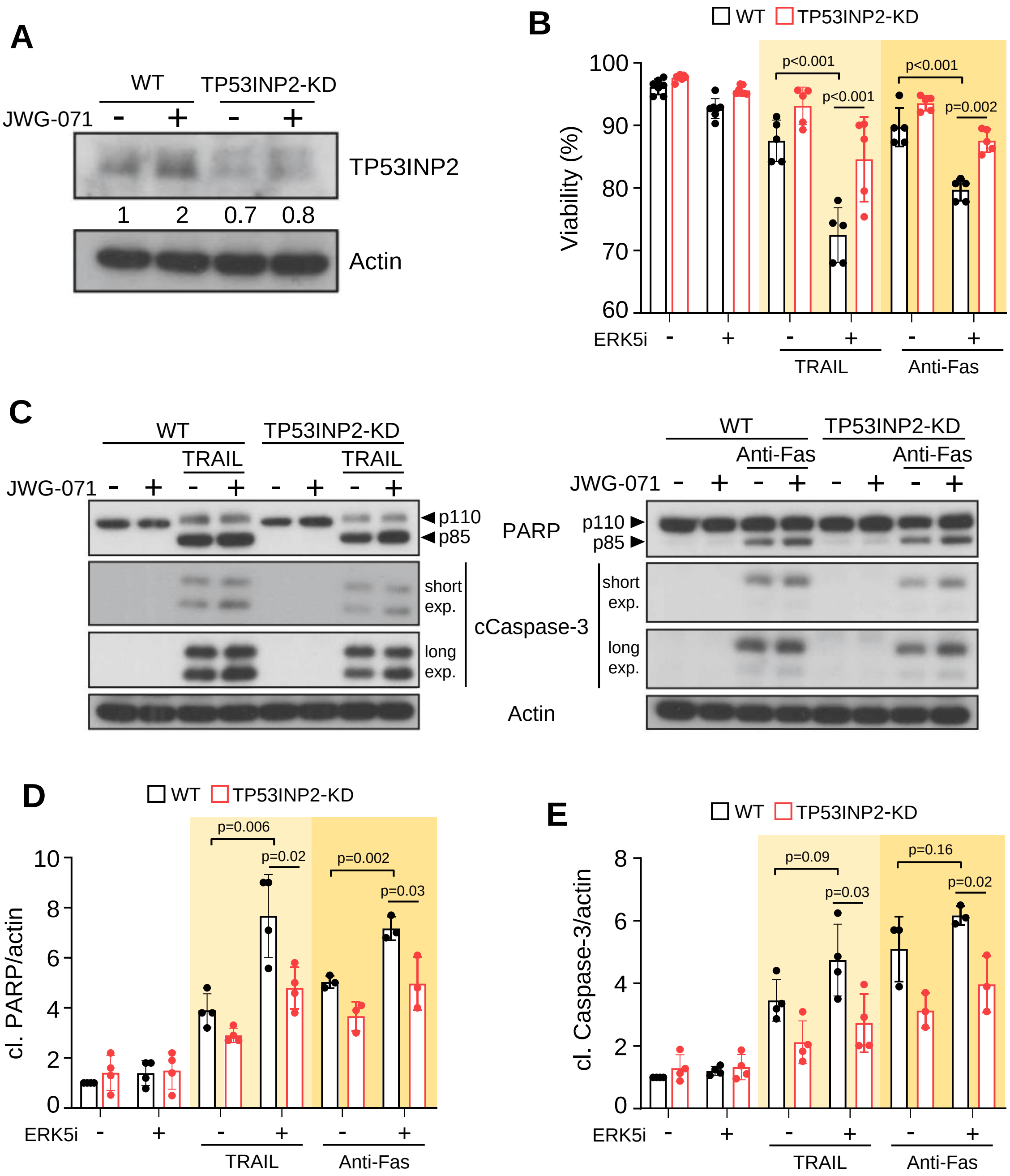

Supplementary Figure 2. ERK5 inhibition sensitizes HeLa cervical cancer cells to DR agonists by increasing TP53INP2 protein levels. A, ERK5 inhibition upregulates TP53INP2 protein levels in HeLa cervical cancer cells. HeLa cells were treated for 24 h with either vehicle or 5 µM JWG-071. TP53INP2 protein levels were determined by immunoblot analysis. **B-E,** TP53INP2 mediates the sensitization to DR agonists exerted by the ERK5i JWG-071 in HeLa cells. **B**, HeLa wild type or TP53INP2-KD cells (were TP53INP2 was knocked-down) were pre-treated 5 µM JWG-071 (16 h), treated with 50 ng/ml TRAIL or 100 ng/ml Anti-Fas activating antibody for 24 h, and cell viability was assessed by annexin V and PI staining. **C**, Activation of apoptosis was measured by immunoblot analysis of cleaved caspase-3 and PARP. Cells were pre-treated with JWG-071 (12 h) and further treated with 50 ng/ml TRAIL or 100 ng/ml Anti-Fas activating antibody for 4 h. **D-E,** Quantification of protein levels of cleaved PARP (**D**) and cleaved caspase-3 (**E**) after TRAIL and anti-Fas treatment. Statistical significance in B, D and E was calculated using two-tailed unpaired t-test. Individual p-values are indicated in each panel.

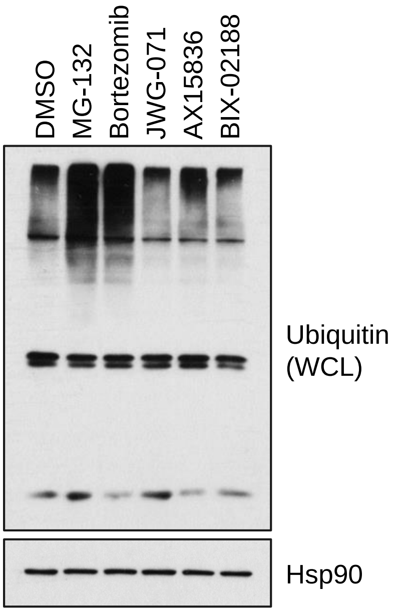

Supplementary Figure 3. MEK5-ERK5 pathway inhibitors do not inhibit the proteasome. Ishikawa cells were treated with proteasome inhibitors (MG-132 or Bortezomib) or ERK5 (JWG-071 and AX15836) and MEK5 (BIX02188) inhibitors for 8 h. Then, cells were lysed and global protein ubiquitylation was determined by immunoblotting for Ubiquitin.

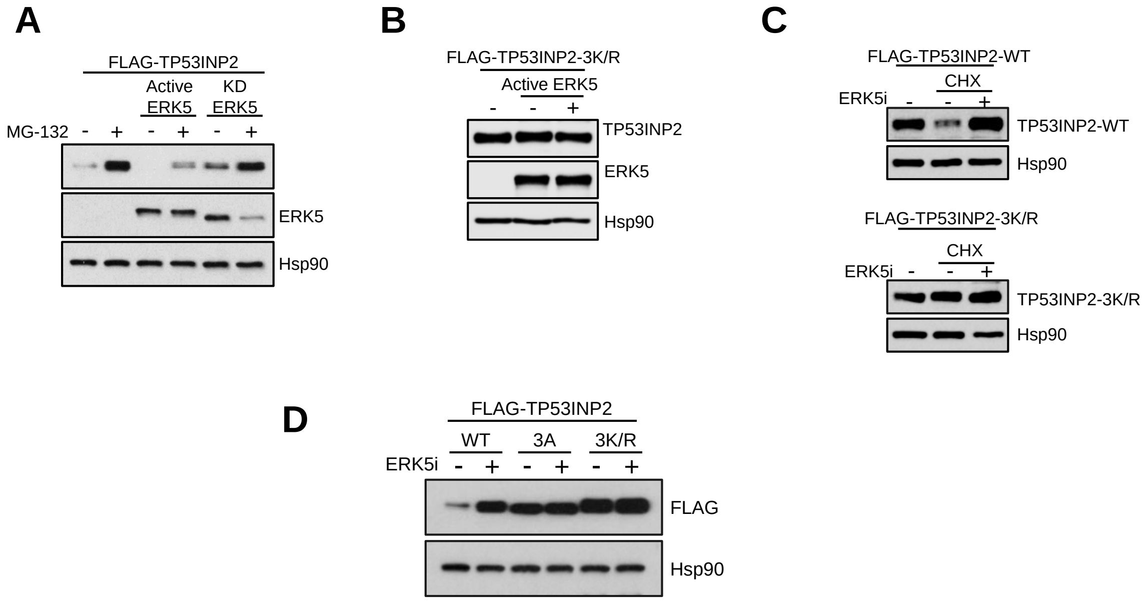

Supplementary Figure 4. Active ERK5 induces TP53INP2 degradation in HEK293T cells. A, Active ERK5 overexpression induces TP53INP2 proteasomal degradation. HEK293T were transfected with vectors encoding for FLAG-TP53INP2 and GST-tagged wild type ERK5 (ERK5-WT) or ERK5 kinase-dead mutant (ERK5-KD), in combination with a vector encoding for a constitutively active form of MEK5 (MEK5-DD). Cells were treated with either vehicle or MG-132 (4 h), and protein levels monitored by immunoblot analysis. **B**, Active ERK5 does not induce proteasomal degradation of the ubiquitylation-deficient mutant TP53INP2-3K/R. Experiment was performed as in **A**. **C**, ERK5 inhibition stabilizes TP53INP2 protein levels. Immunoblot analysis of HEK293 cell lysates overexpressing wild type or ubiquitylation-deficient mutant, treated with ERK5i and the protein synthesis inhibitor cyclohexamide (CHX) for 8 hours. **D**, The TP53INP2-3A and TP53INP2-3K/R mutants have higher protein expression than wild-type TP53INP2, and ERK5i does not affect protein levels of TP53INP2-3A and TP53INP2-3K/R mutants. Immunoblot analysis.

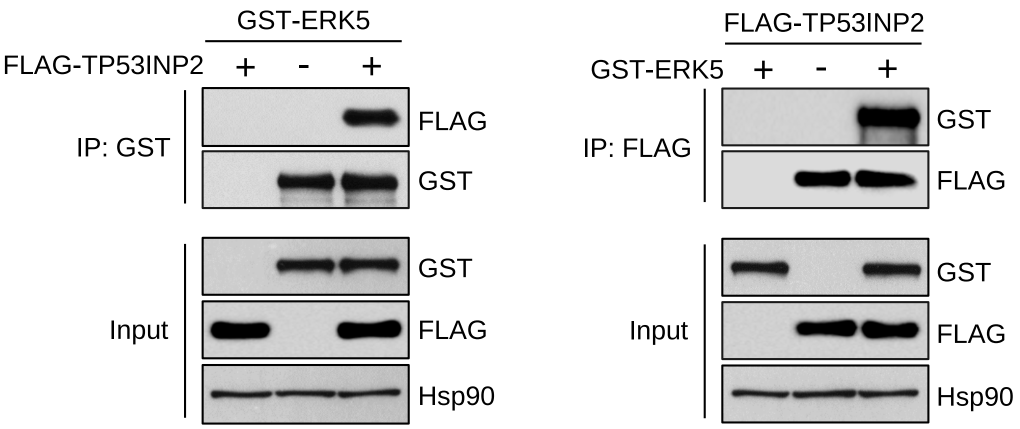

Supplementary Figure 5. ERK5 interacts with TP53INP2. HEK293T cells were transfected with a vector encoding for FLAG-tagged TP53INP2 alone or together with GST-tagged ERK5. Forty-eight hours later, cells were lysed in NP40 buffer and FLAG-TP53INP2 or GST-ERK5 were affinity-purified using ANTI-FLAG® M2 Affinity Beads or glutathione-sepharose, respectively. Immune complexes were immunoblotted for ERK5 and TP53INP2.

A

Sequence	#PSMs	Modifications	MH+ [Da]	ERK5	Empty
SKNQS**S**FIYQPCQR	10	S6(Phospho); C12(Carbamidomethyl)	1822,79	x	x
LSSLFFSTP**S**PPEDPDCPR	20	S10(Phospho); C17(Carbamidomethyl)	2228,96	x	x
R**S**KNQSSFIYQPCQR	1	S2(Phospho); C13(Carbamidomethyl)	1978,90		x
NQS**S**FIYQPCQR	3	S4(Phospho); C10(Carbamidomethyl)	1607,67	x	x
LSSLFFS**T**P**S**PPEDPDCPR	7	T8(Phospho); S10(Phospho); C17(Carbamidomethyl)	2308,93	x	x

B

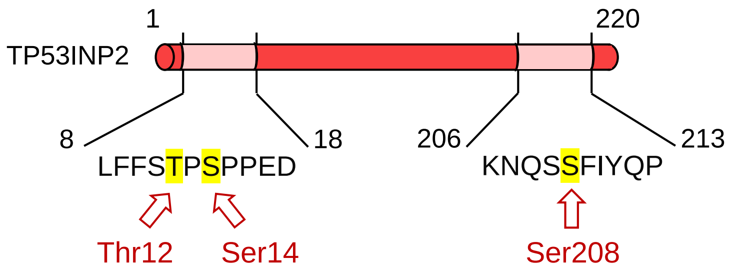

Supplementary Figure 6. TP53INP2 protein is constitutively phosphorylated in Thr14, Ser14 and Ser208 in cells. A. Protein TP53INP2 (Q81HX6) phosphorylated peptides identified by LC-MS/MS, and the graphical representation of its corresponding residues in the original TP53INP2 protein sequence (**B**).

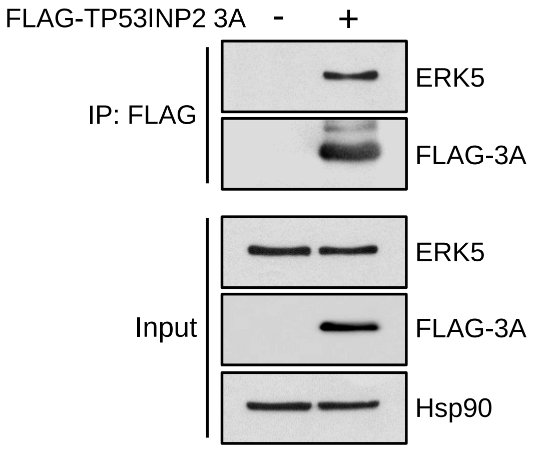

Supplementary Figure 7. The TP53INP2 phospho-deficient mutant retains the ability to interact with ERK5. HEK293T cells were transfected with a vector encoding for FLAG-tagged TP53INP2-3A. Forty-eight hours later, cells were lysed in NP40 buffer and FLAG-TP53INP2 was affinity-purified using ANTI-FLAG® M2 Affinity Beads. Immune complexes were immunoblotted for endogenous ERK5 and TP53INP2.

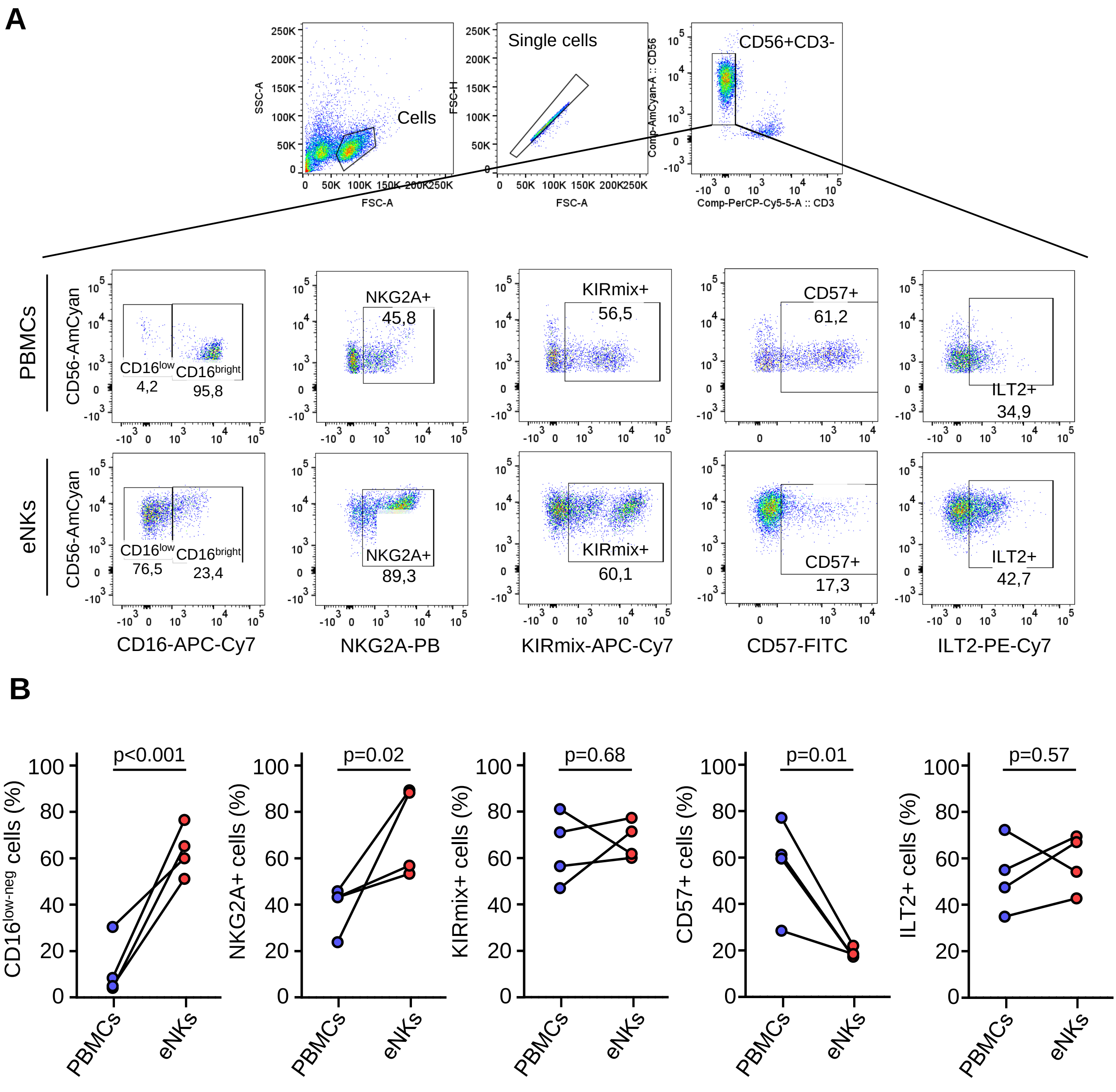

Supplementary Figure 8. Comparative phenotypic analysis of fresh and eNK cells. A-B. eNK cells and PBMCs from four healthy donors were stained for different NK cell surface markers (NKG2A, KIR, ILT2, CD16, CD57), analyzed and gated on forward and side scatter (FSC/SSC) and CD56+ /CD3- cells. Representative flow cytometry dot plots **(A)** and graphs of the results from samples of four donors **(B)** are depicted.

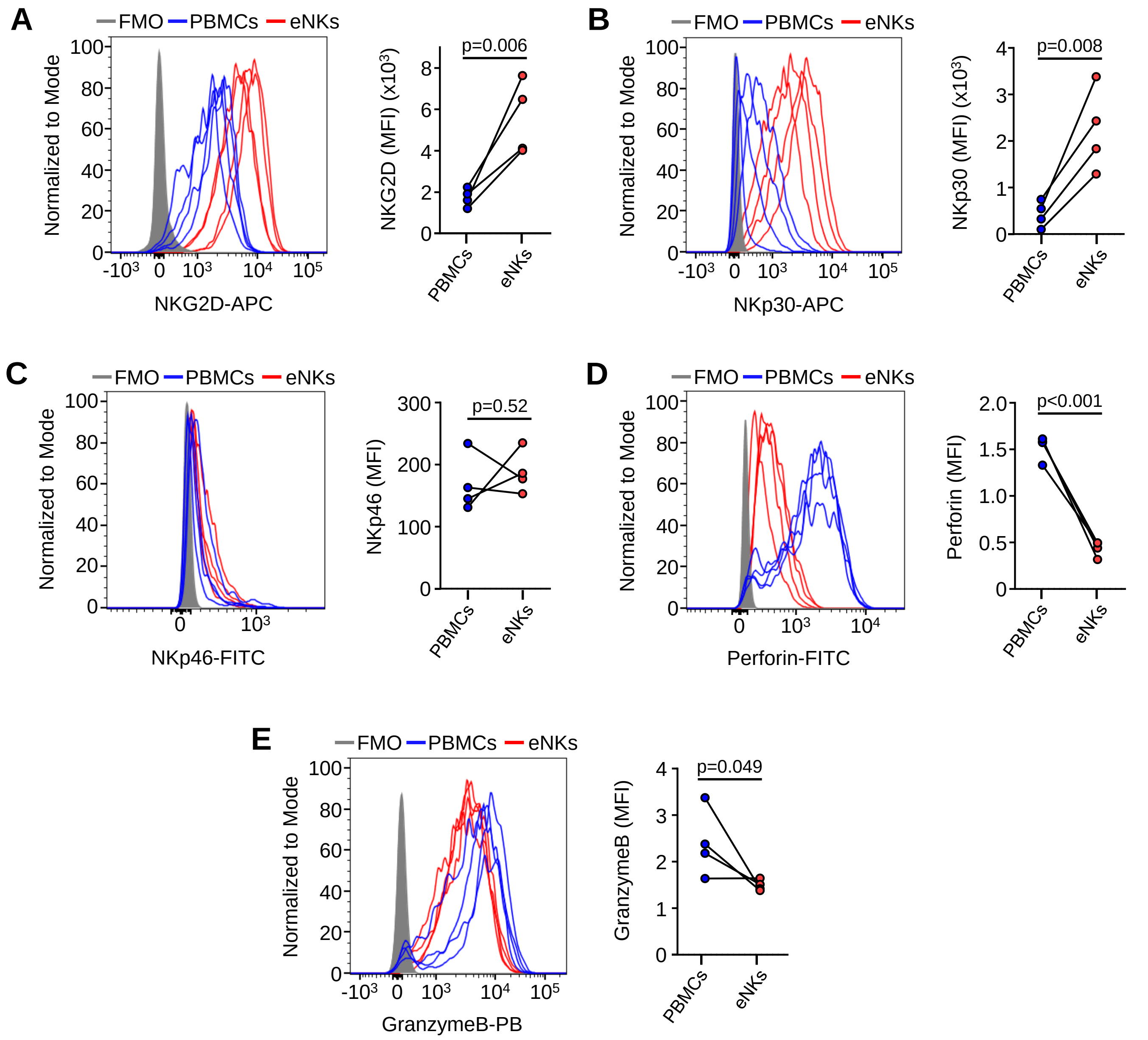

Supplementary Figure 9. Expression of activating receptors and intracellular cytotoxic mediators by fresh and eNK cells. eNK cells and PBMCs from four healthy blood donors were stained for surface markers including activating receptors NKG2D (**A**), NKp30 (**B**) and NKp46 (**C**) and analyzed by flow cytometry. In parallel, samples were fixed, permeabilized and stained for cytotoxic intracellular proteins Perforin (**D**), GranzymeB (**E**). Cells were gated on forward and side scatter (FSC/SSC), and NK cells were gated as CD56+ /CD3- cells.

Patient	Age	Histology	Grade	FIGO	p53	MSH6	PMS2	POLE	TCGA
440	75	Endometrioid	2	II	WT	WT	Mut	WT	MSI
1297	69	Endometrioid	3	Ib	Mut	WT	Mut	Mut	POLE

Supplementary Figure 10. Clinicopathological features of endometrial cancer patients used for PDX-Os generation. WT: wild-type; MSH6: MutS Homolog 6; PMS2: PMS1 Homolog 2; POLE: polymerase epsilon; MSI: microsatellite instability.
