# Supplementary material for: MAP kinase ERK5 modulates cancer cell sensitivity to extrinsic apoptosis induced by death-receptor agonists
Source: Cell Death Dis. 2023 Nov 2;14(11):715. doi: 10.1038/s41419-023-06229-6 (PMC10622508; doi:10.1038/s41419-023-06229-6)
Supplement: Supplementary file 6 — Original Data File [file 41419_2023_6229_MOESM6_ESM.pdf]

FIGURE 1E TRAIL

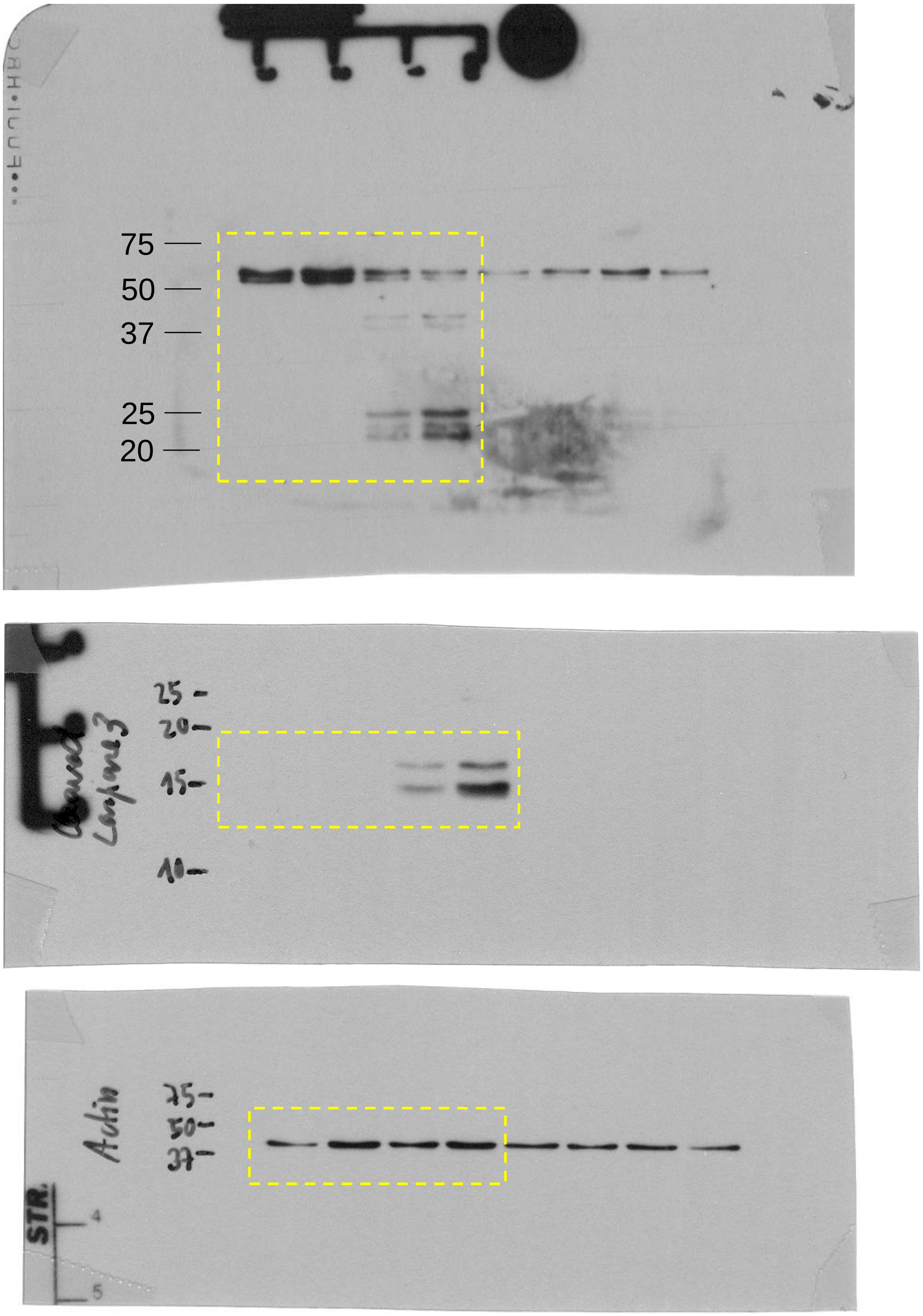

FIGURE 1E TNFa

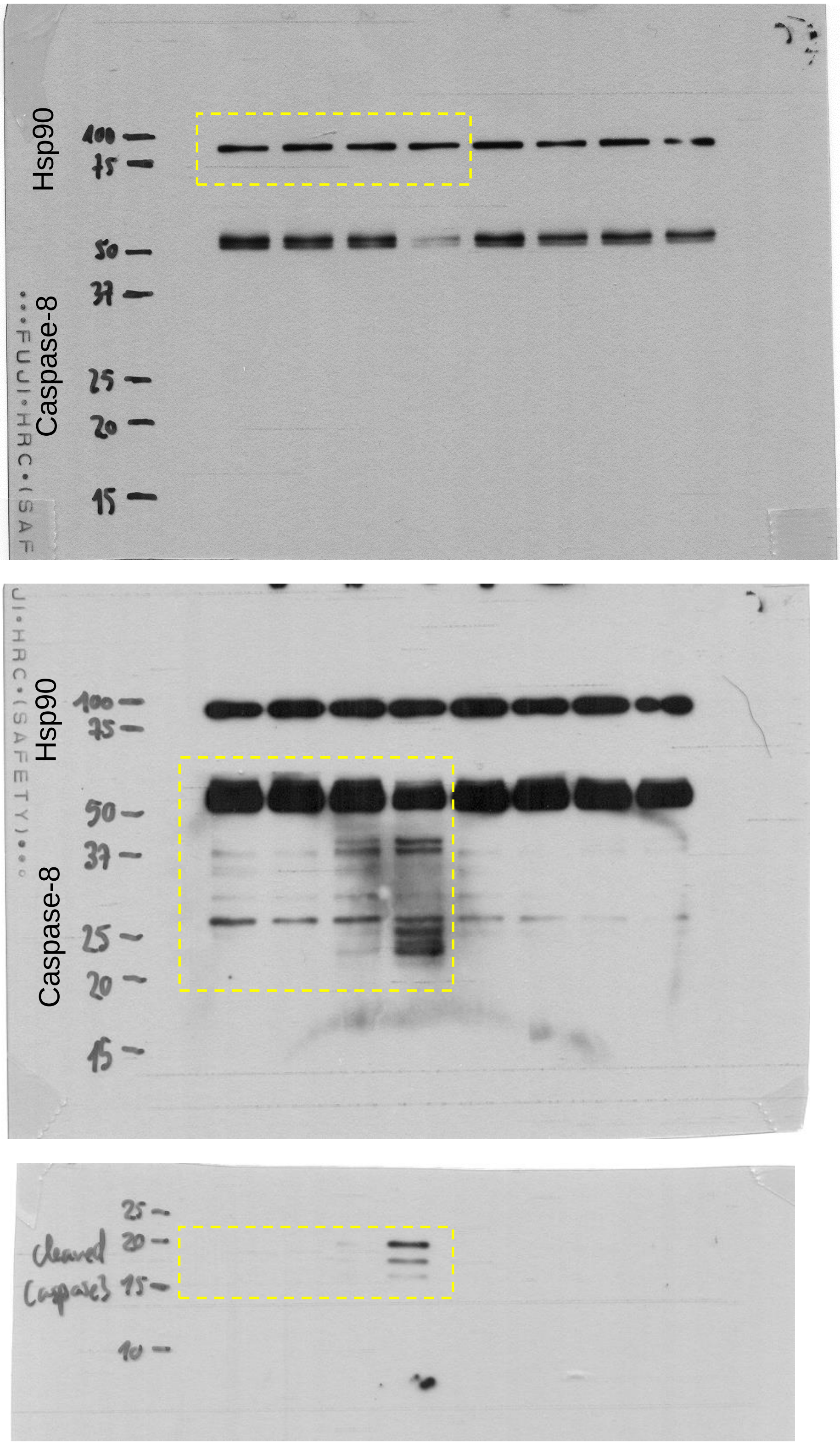

FIGURE 1E anti-Fas

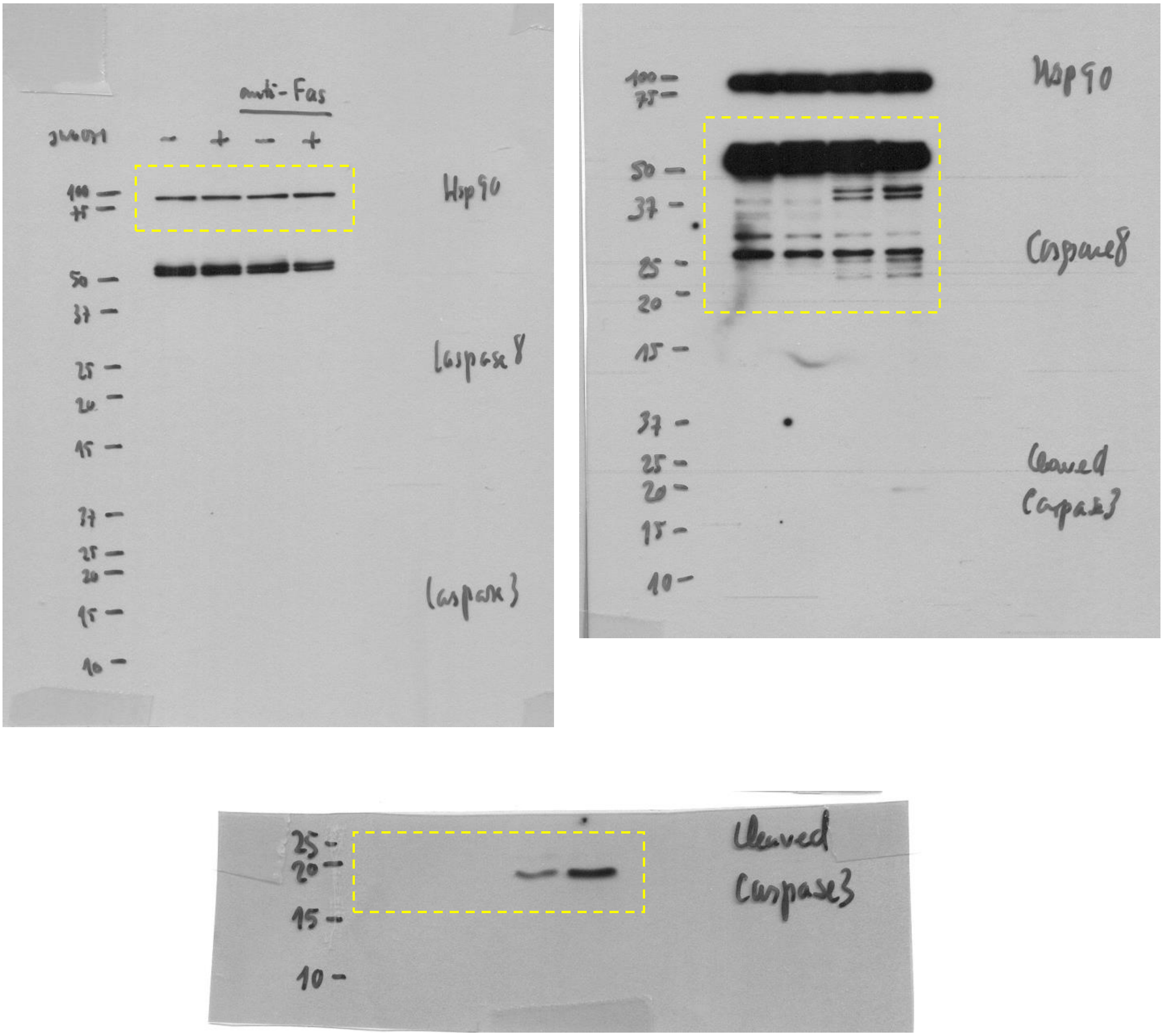

FIGURE 2B AN3CA

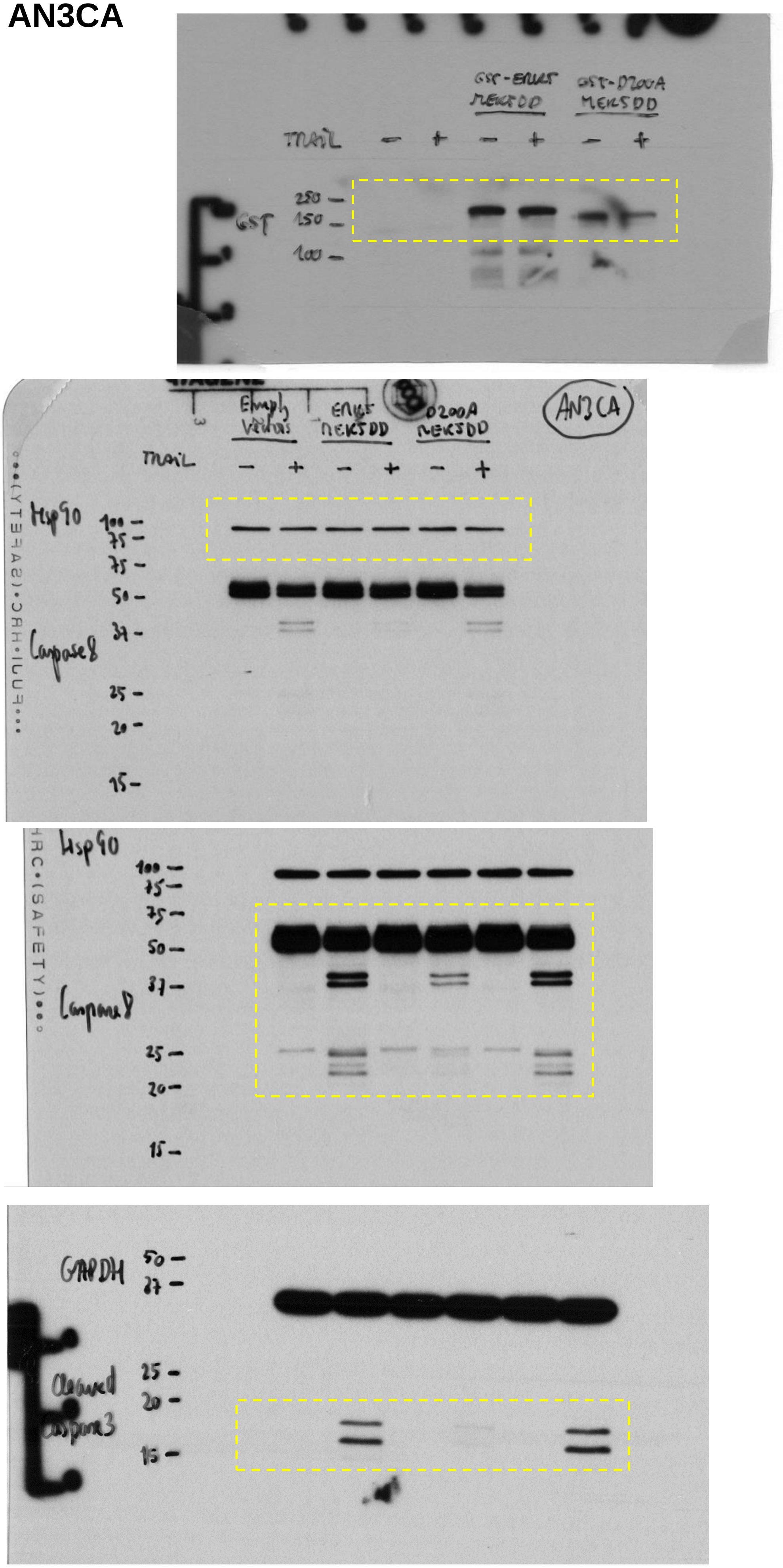

FIGURE 2C Ishikawa

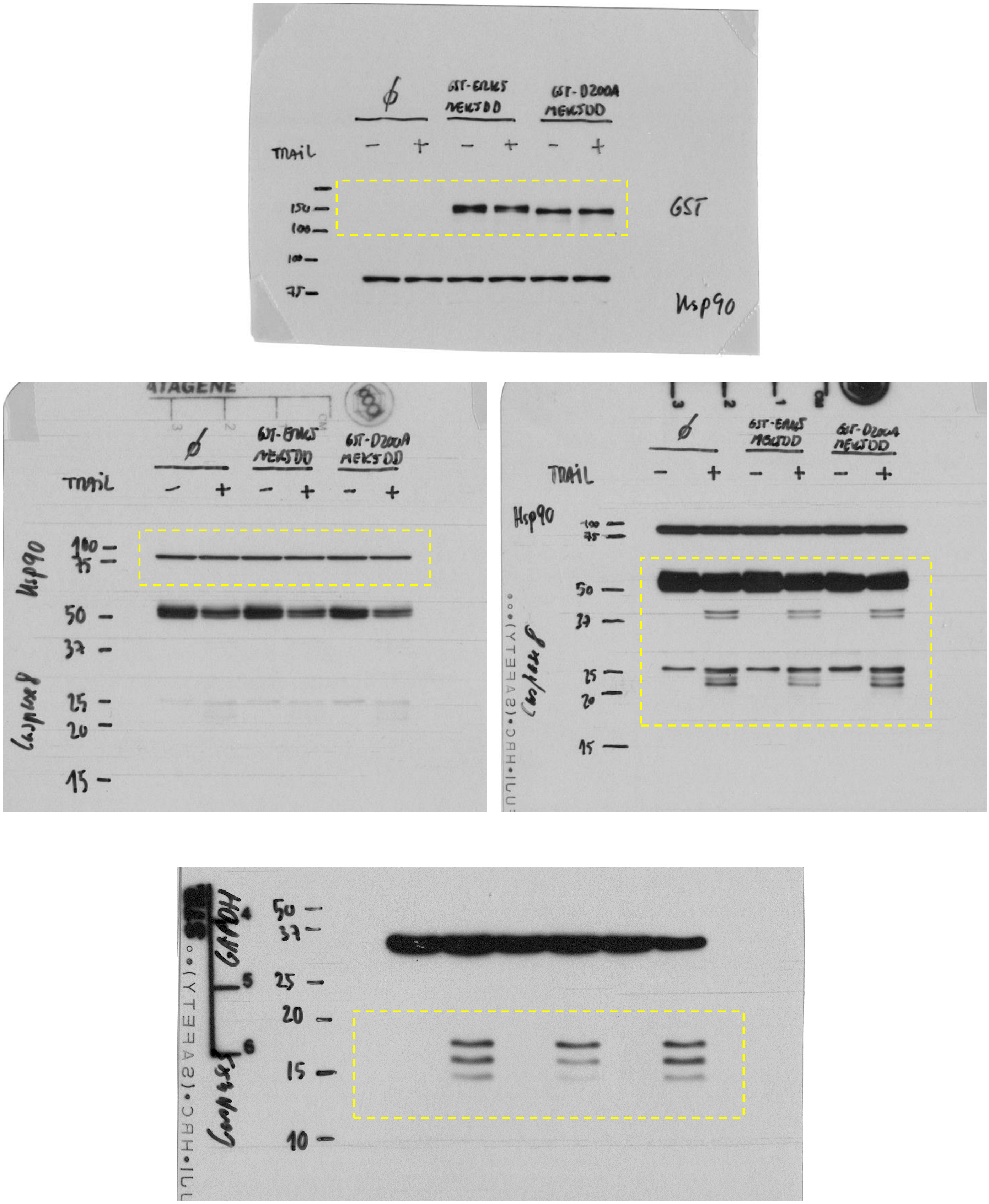

FIGURE 3B TRAIL

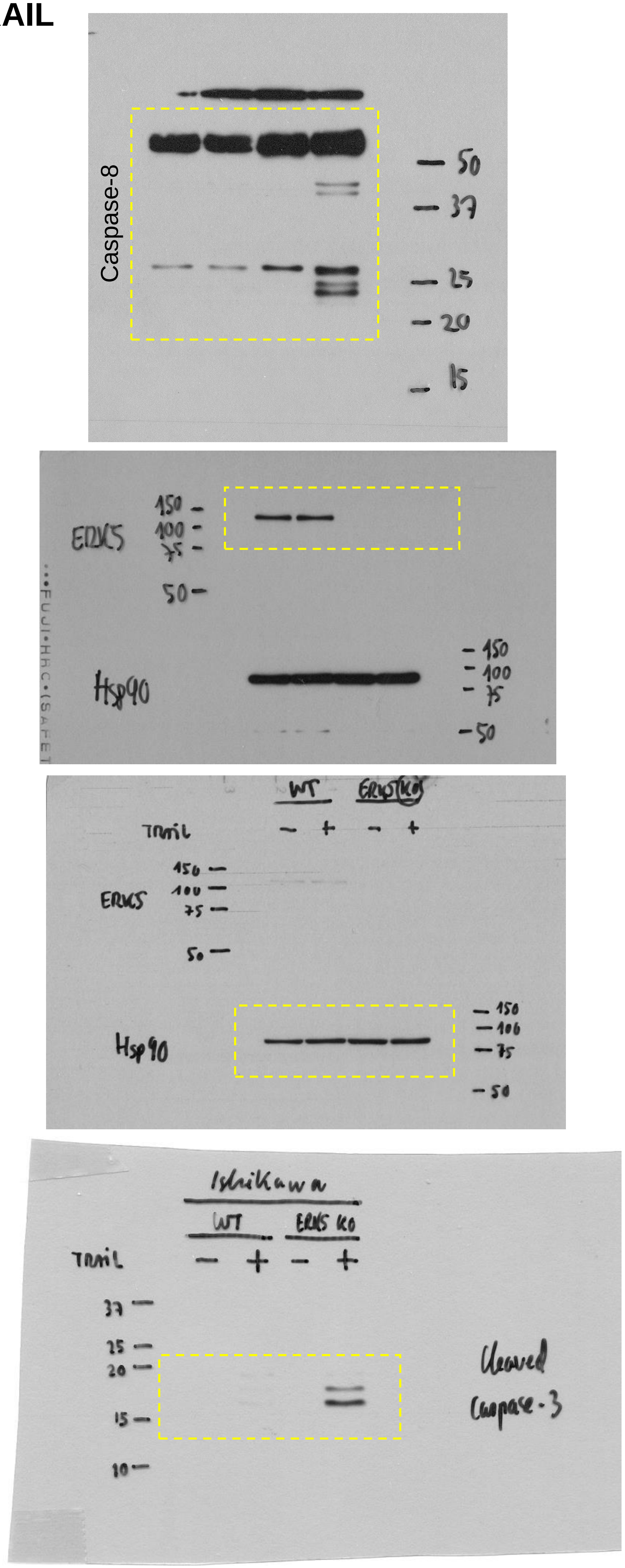

FIGURE 3B TNFa

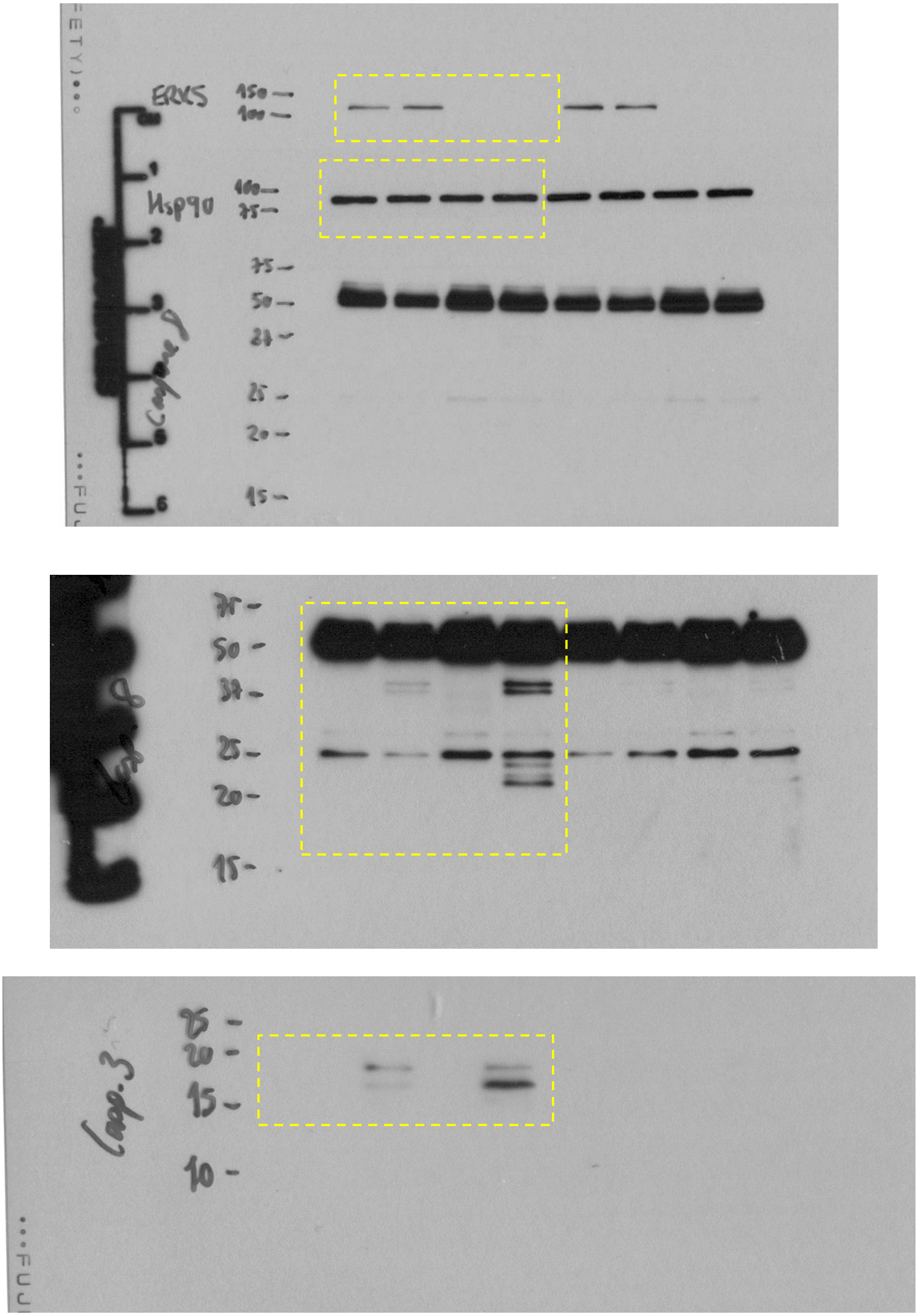

FIGURE 3B anti-Fas

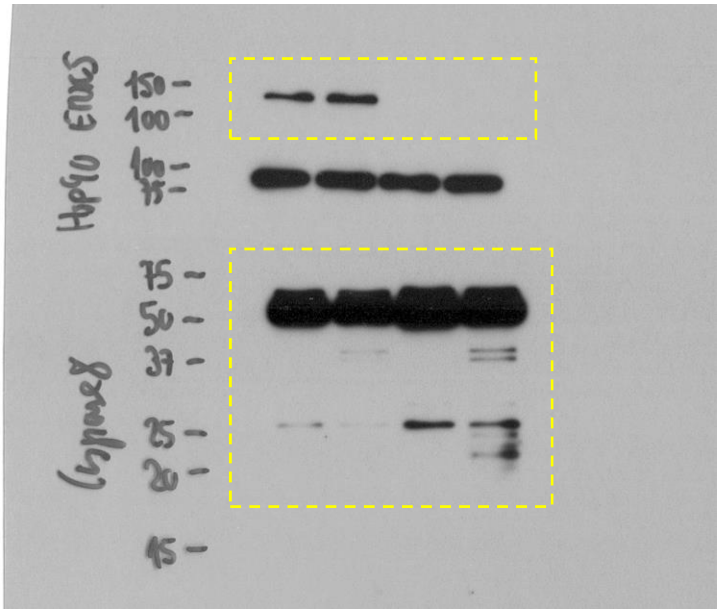

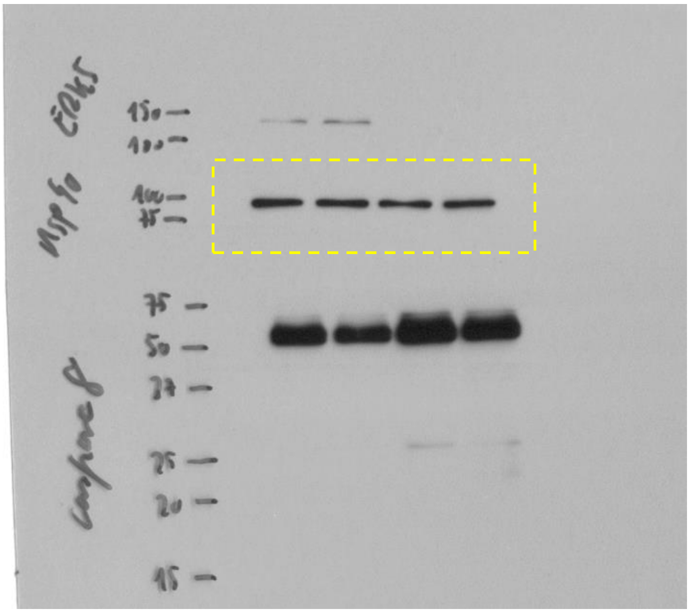

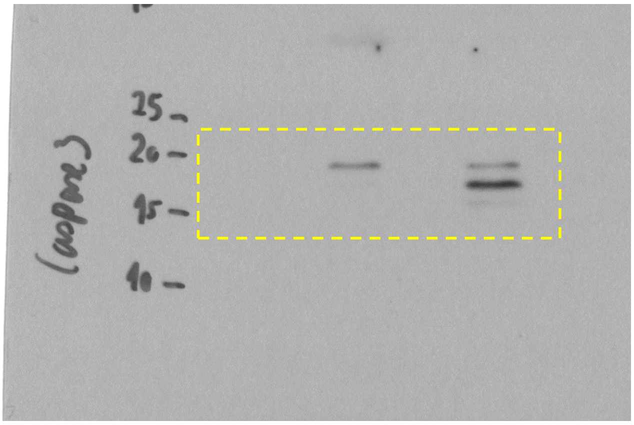

FIGURE 3C TRAIL

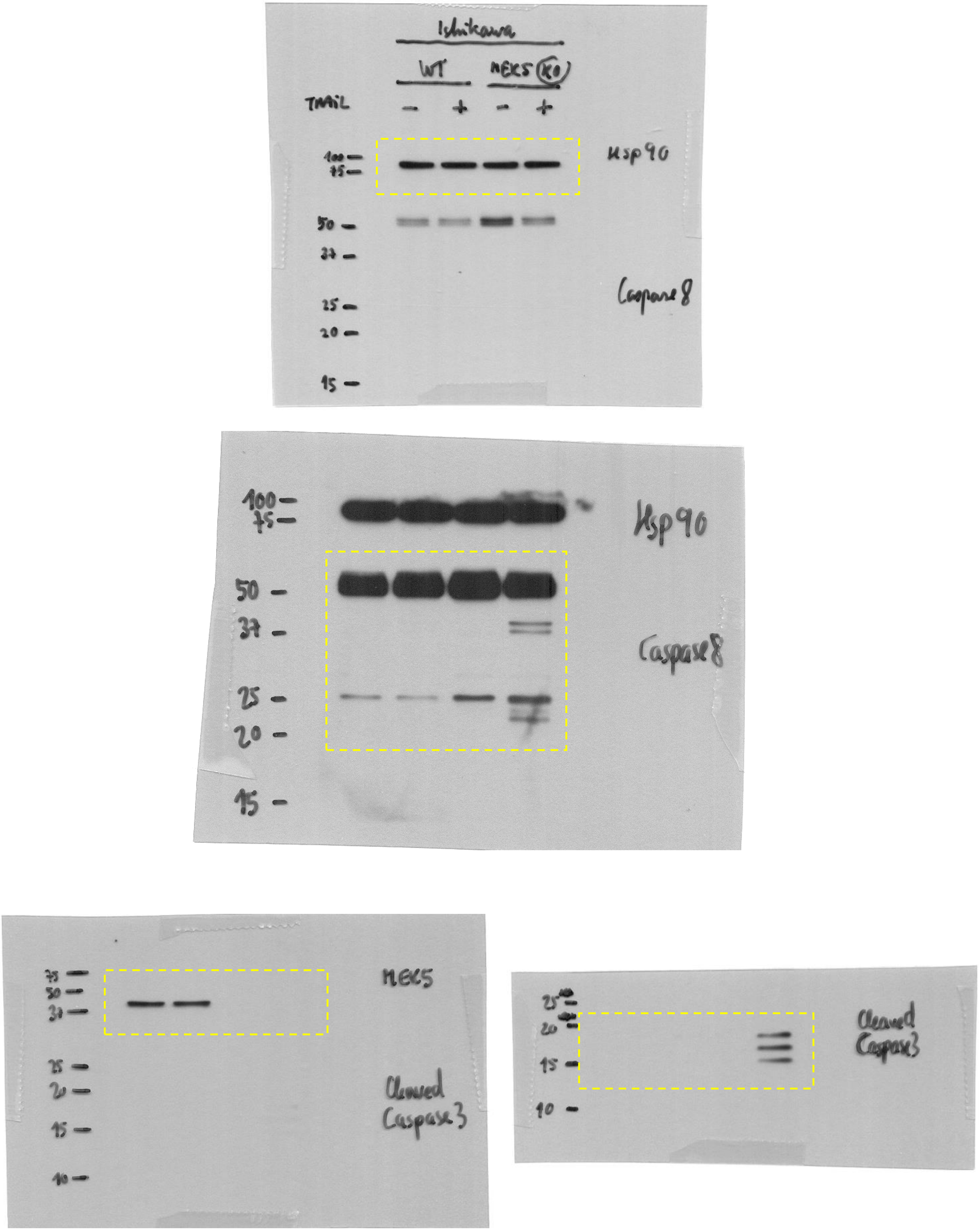

FIGURE 3C TNFa

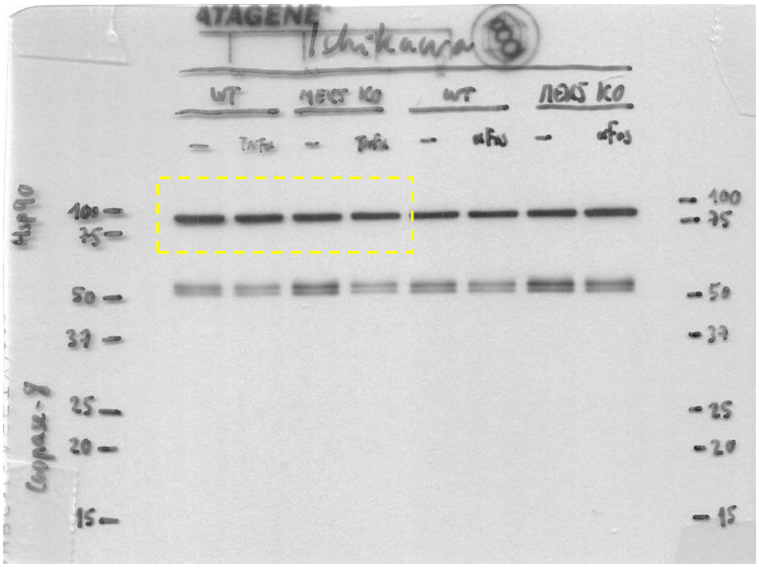

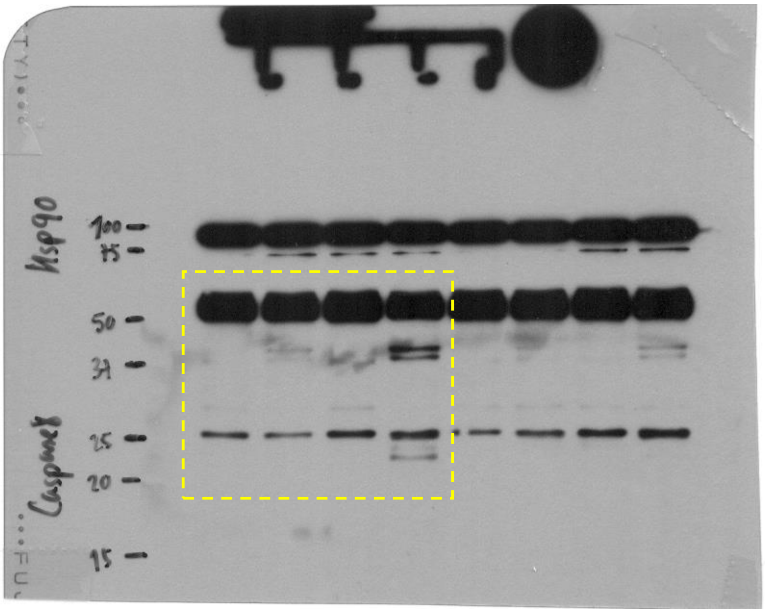

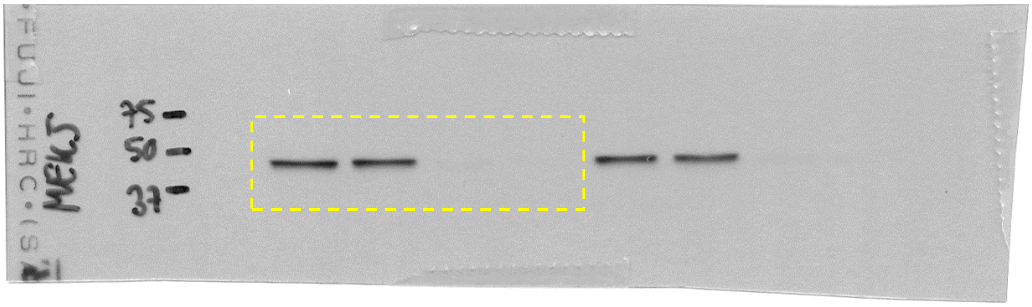

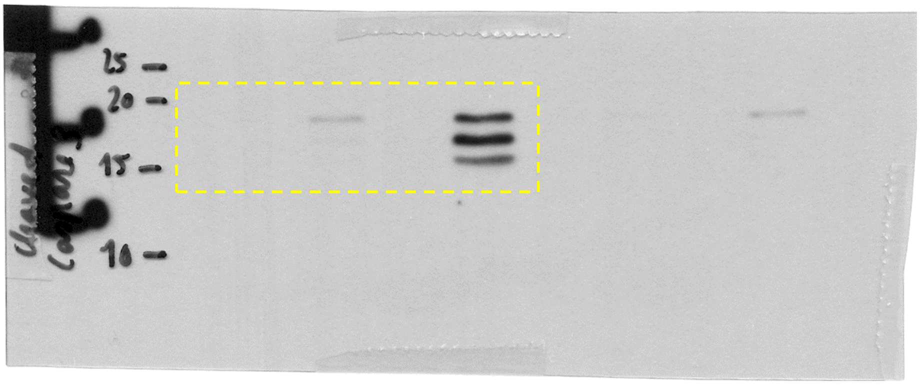

FIGURE 3C anti-Fas

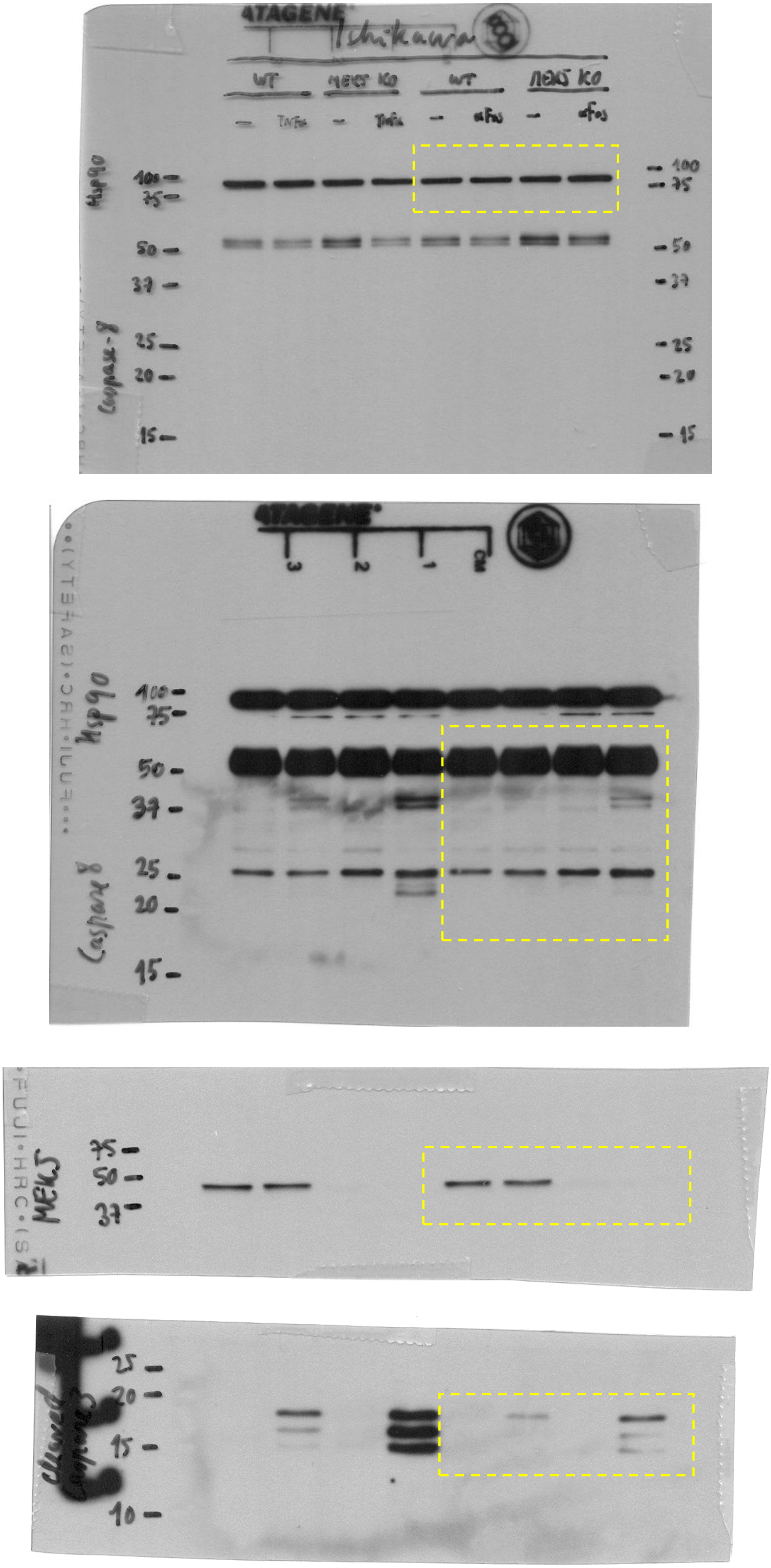

FIGURE 5A Ishikawa

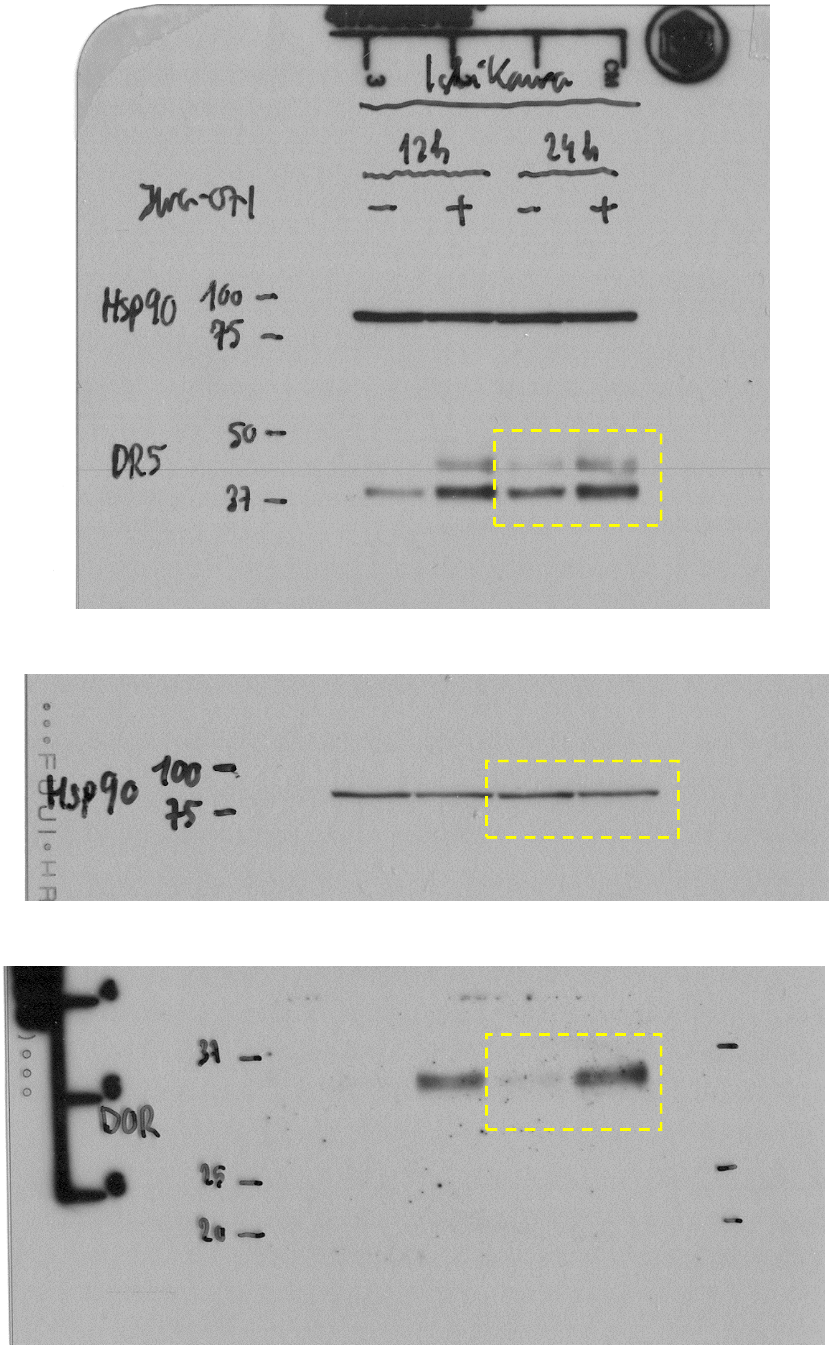

FIGURE 5A ARK1

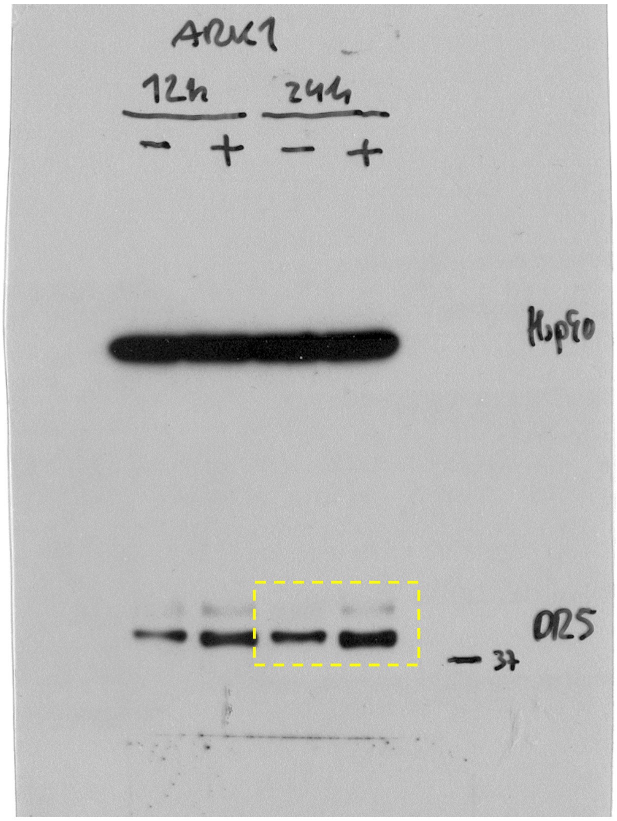

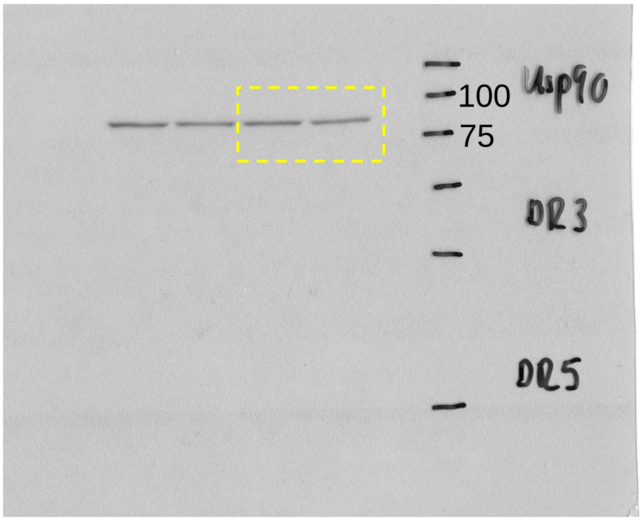

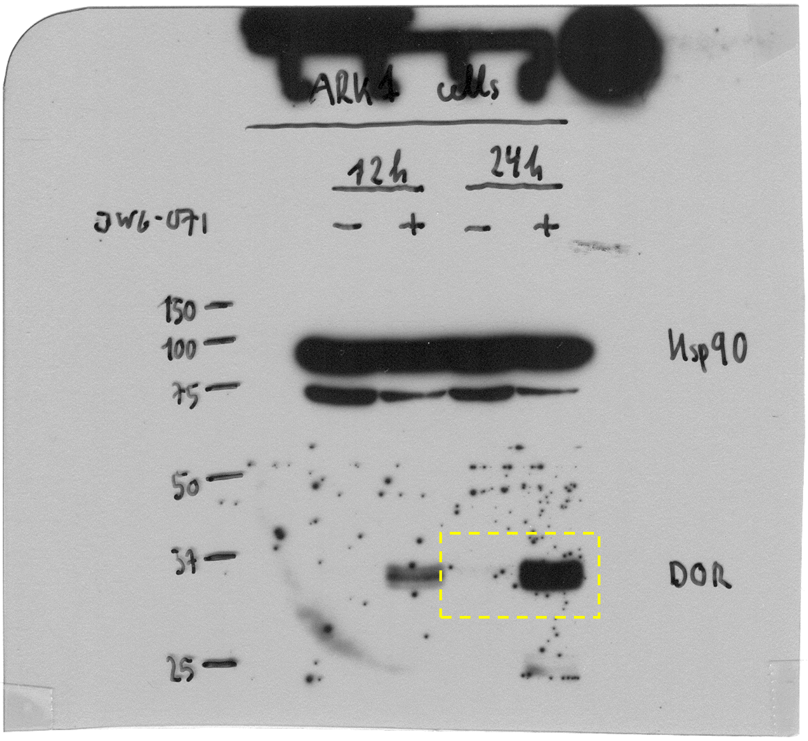

FIGURE 5A ARK2

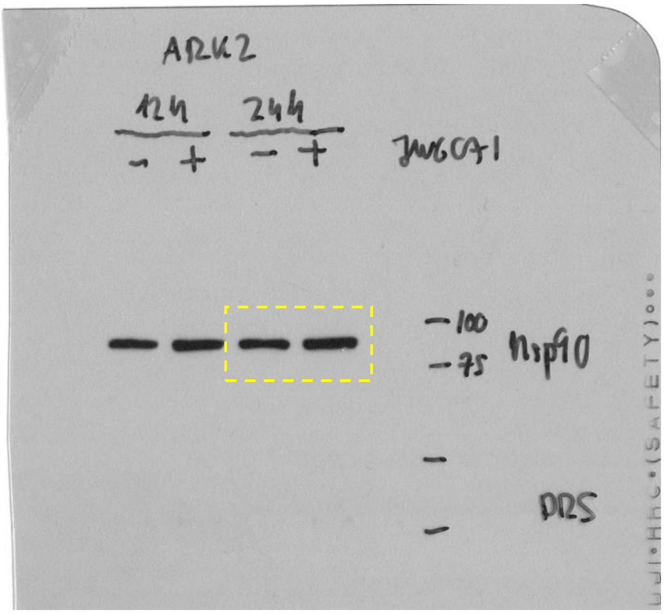

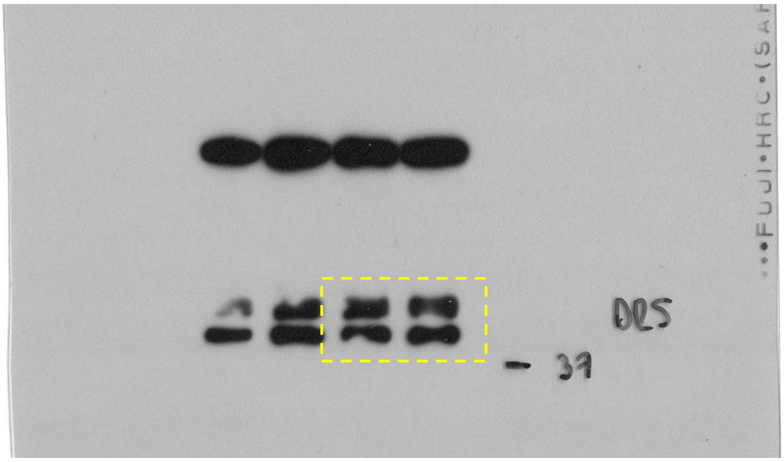

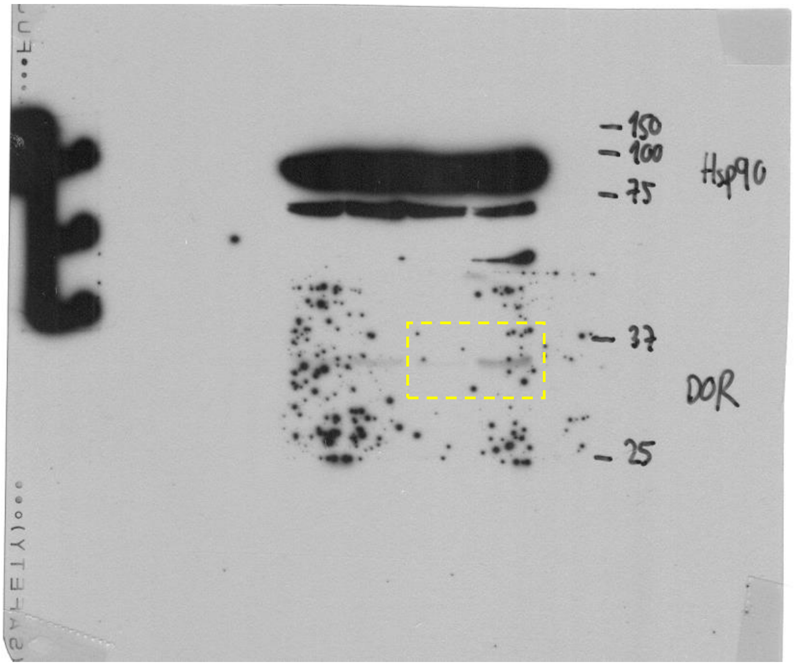

FIGURE 5A AN3CA

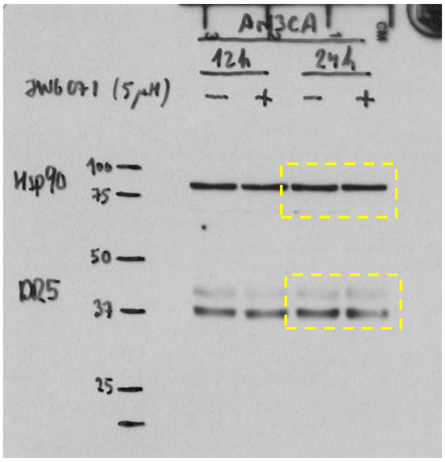

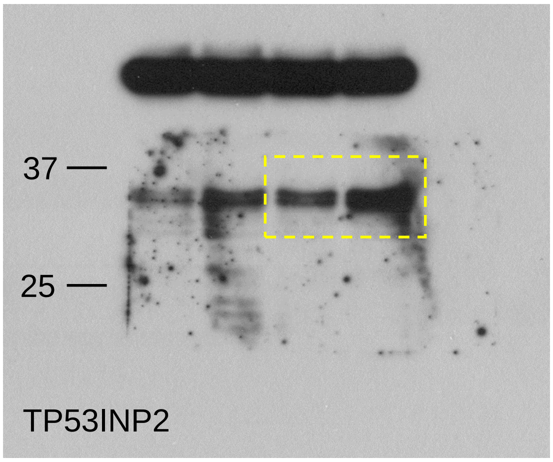

FIGURE 5B ERK5 KO

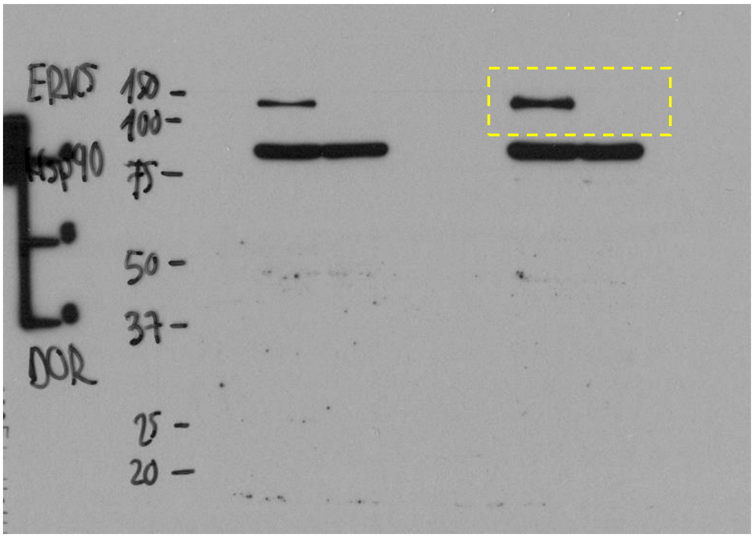

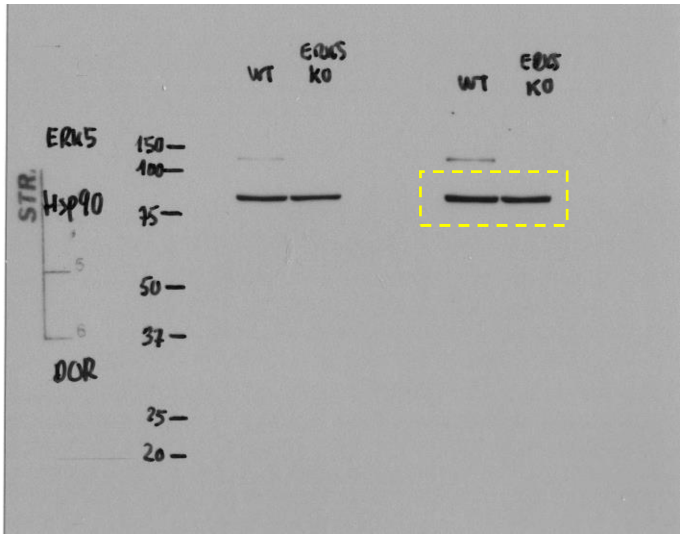

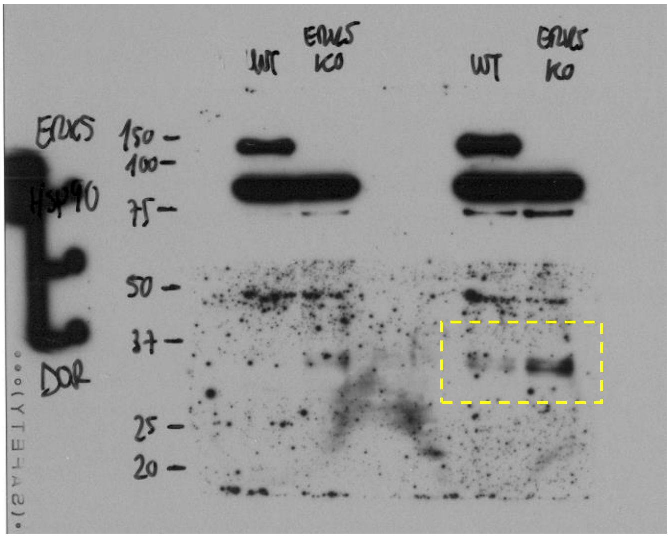

FIGURE 5B MEK5 KO

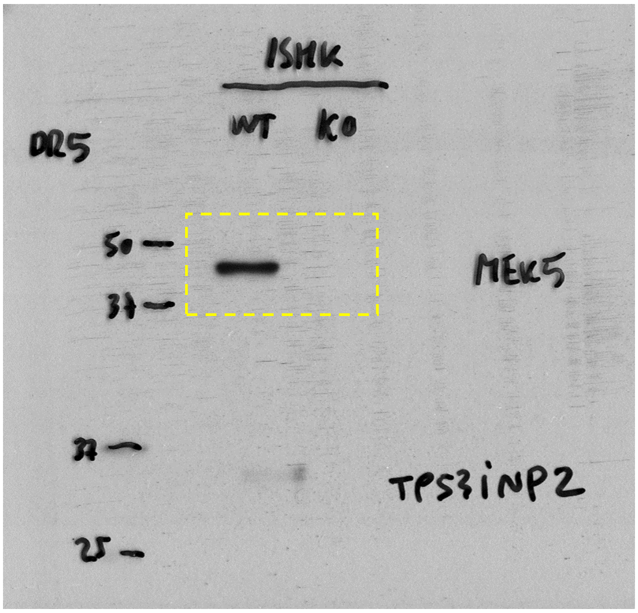

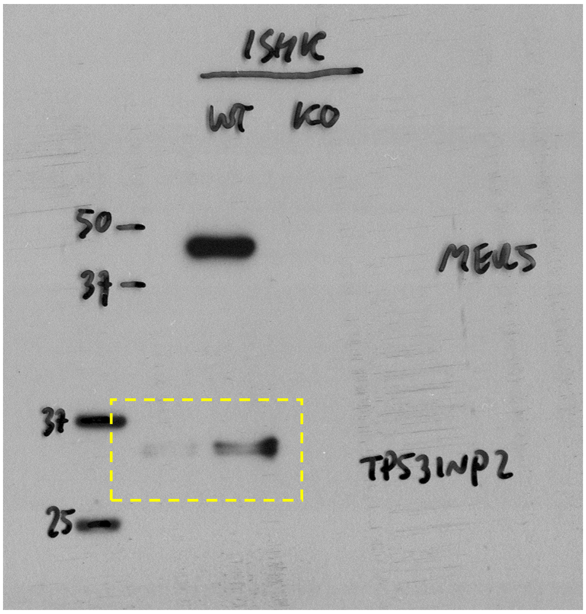

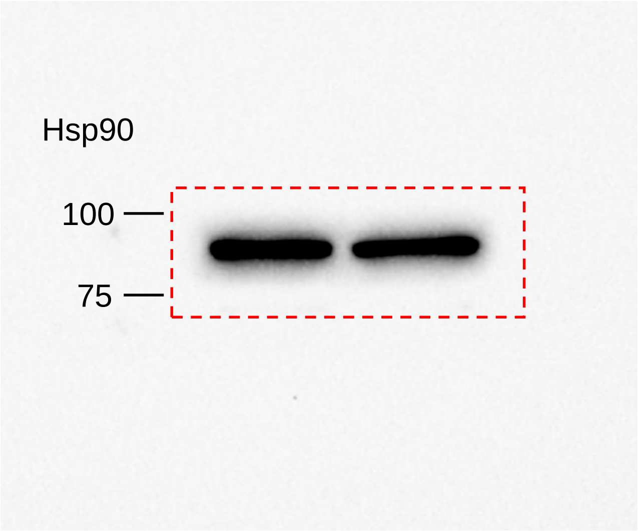

FIGURE 5C

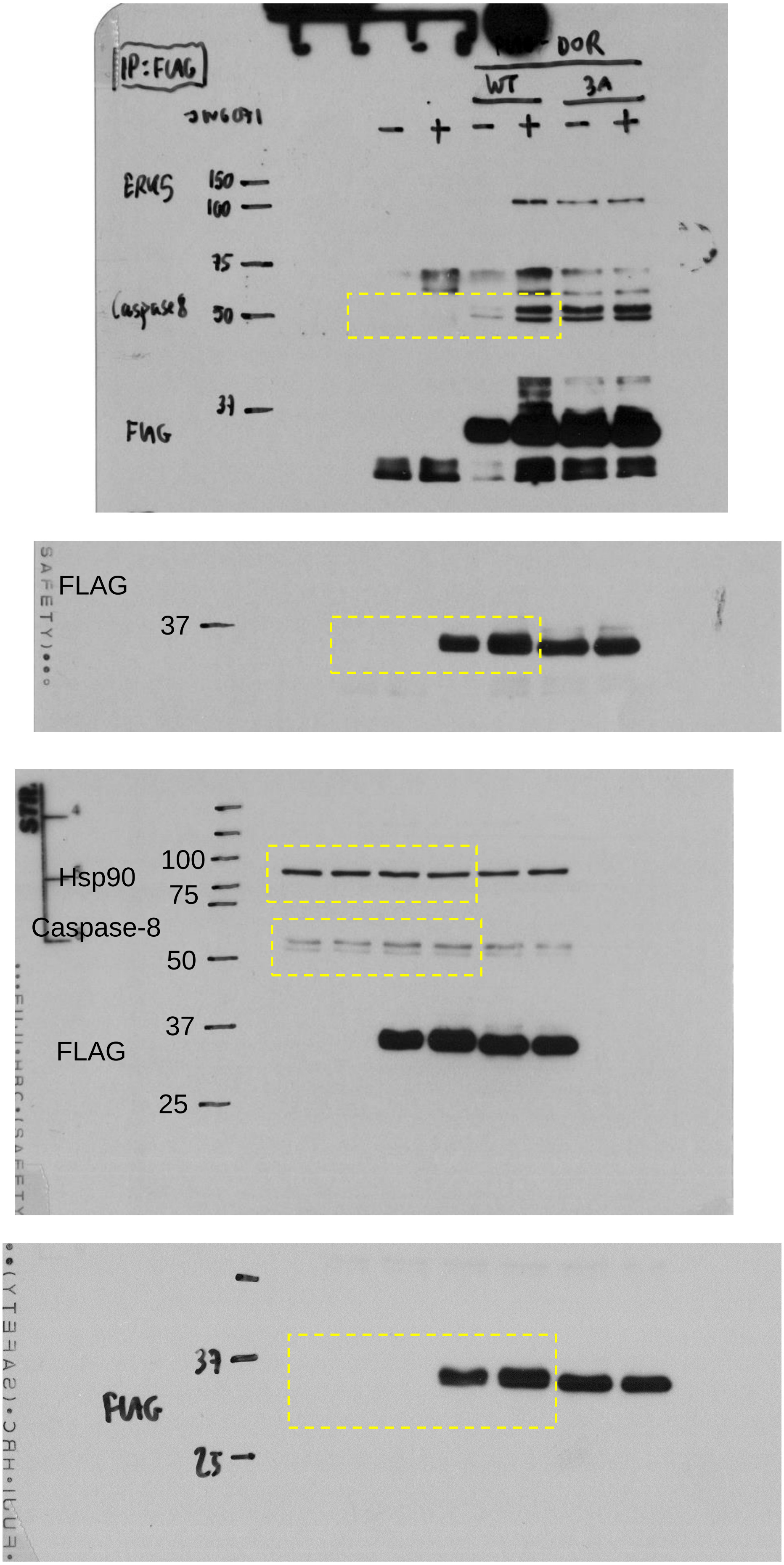
IP:FLAG
WT
3A
ERK5
150
100
75
Caspase8
50
37
FLAG
FLAG
37
100
Hsp90
75
Caspase-8
50
37
FLAG
25
37
FLAG
25

FIGURE 5F TRAIL

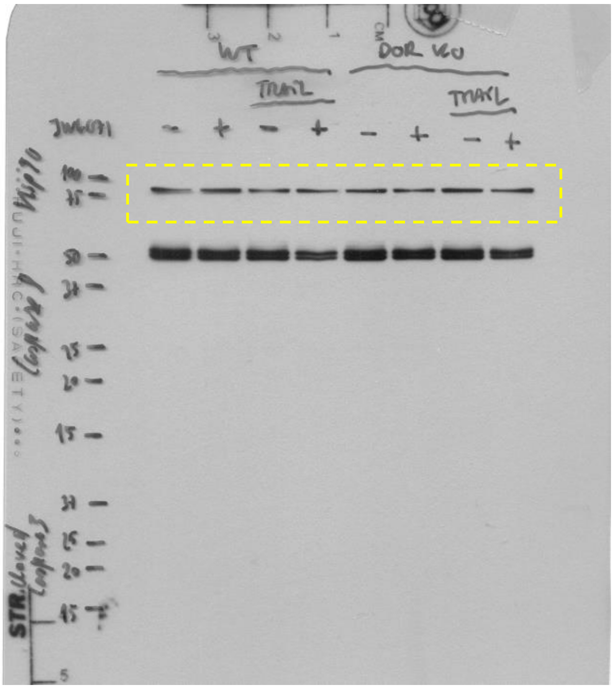

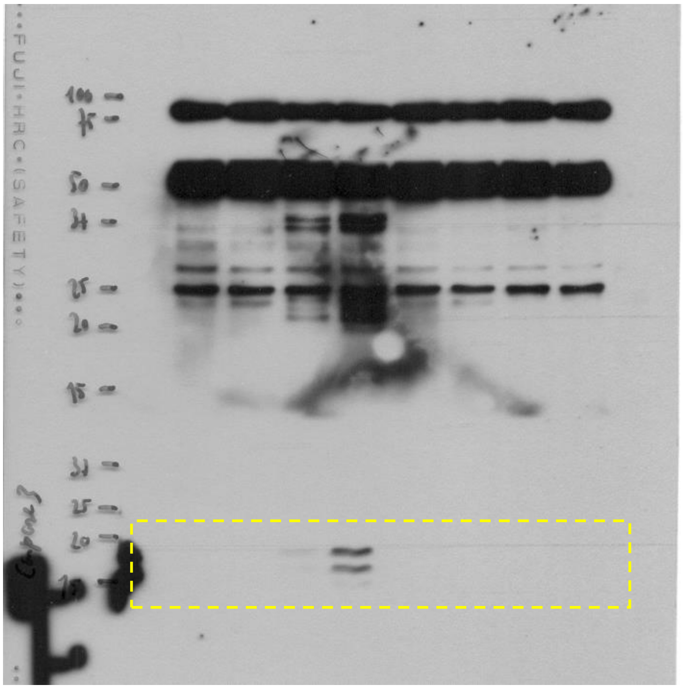

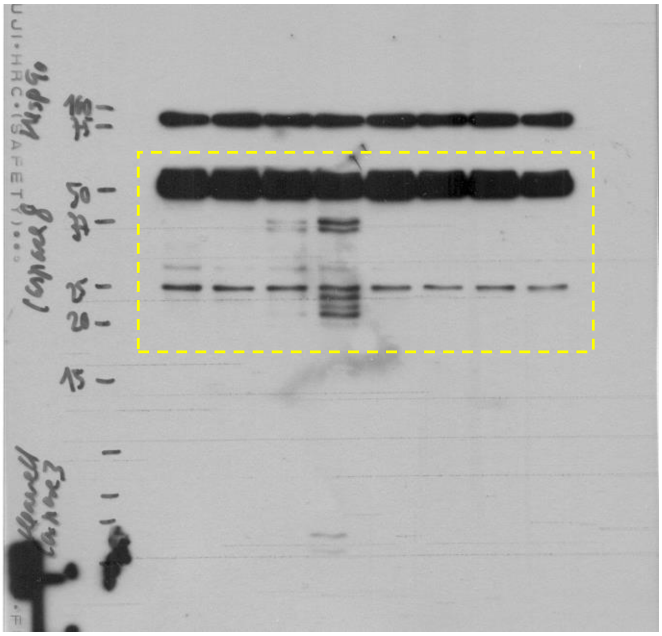

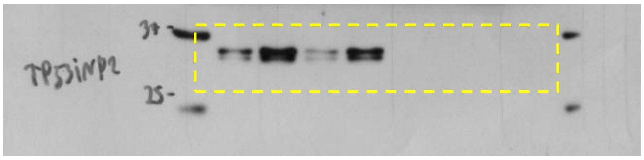

FIGURE 5F TNFa

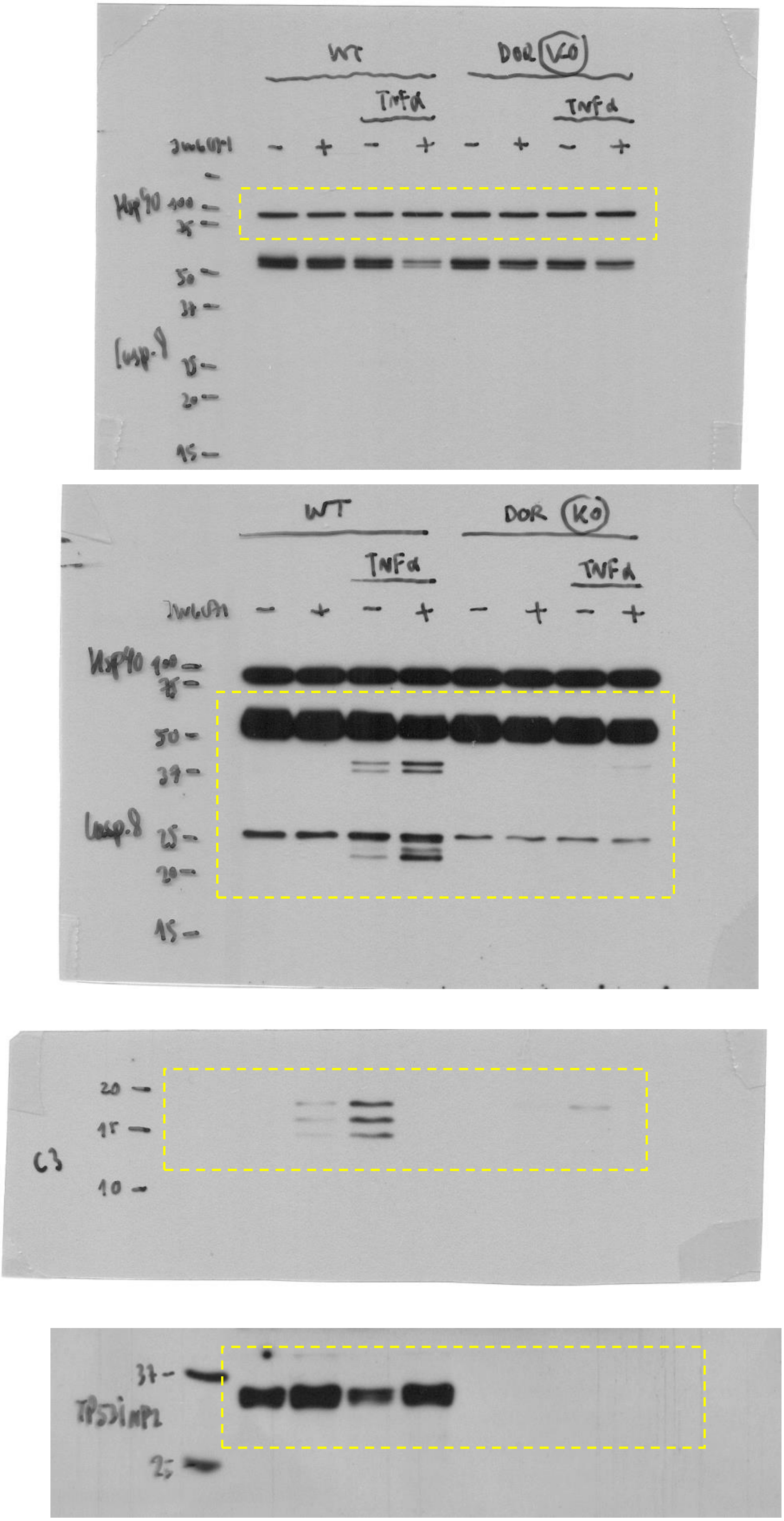

FIGURE 5F anti-Fas

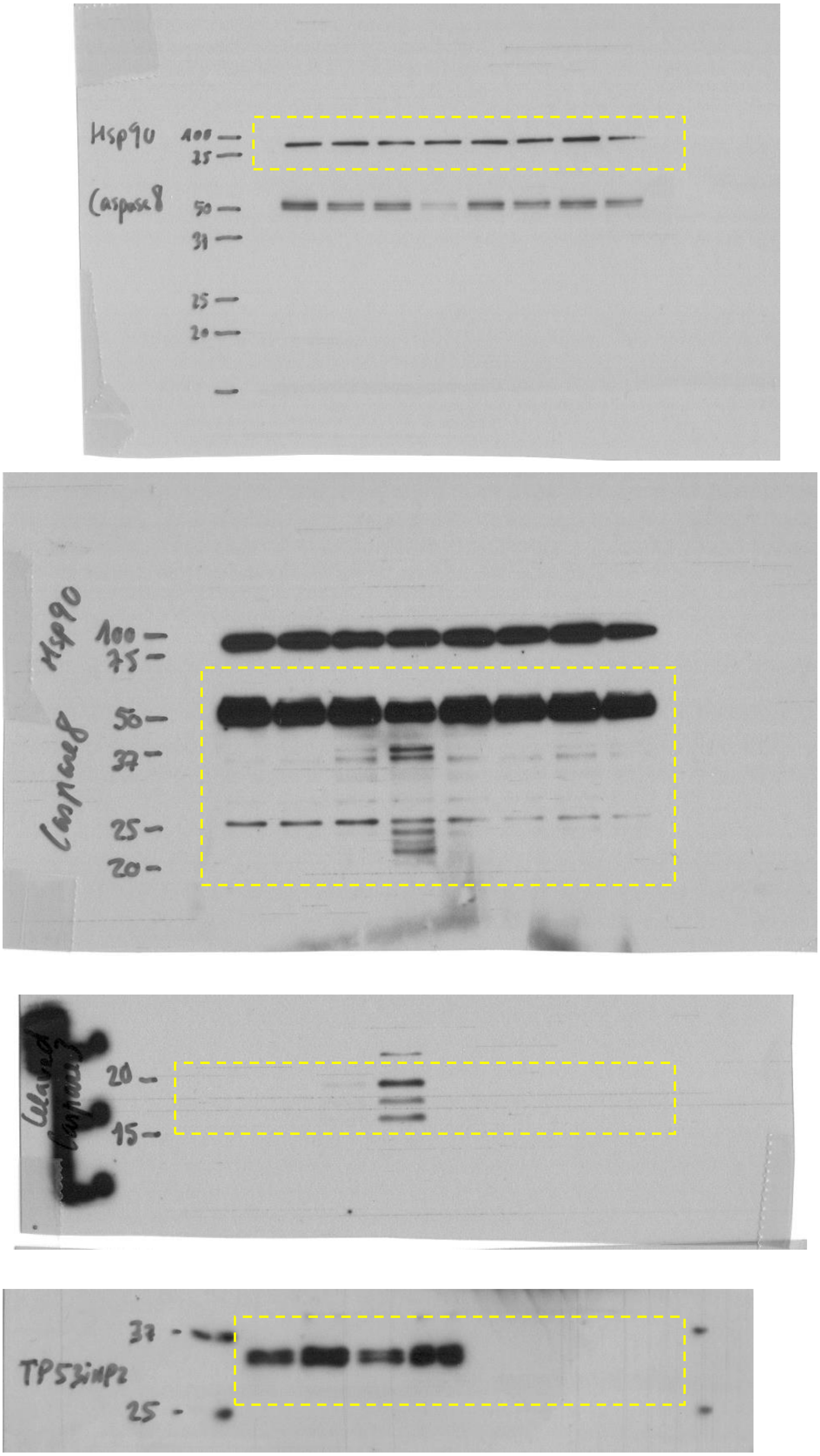

FIGURE 6A Ishikawa

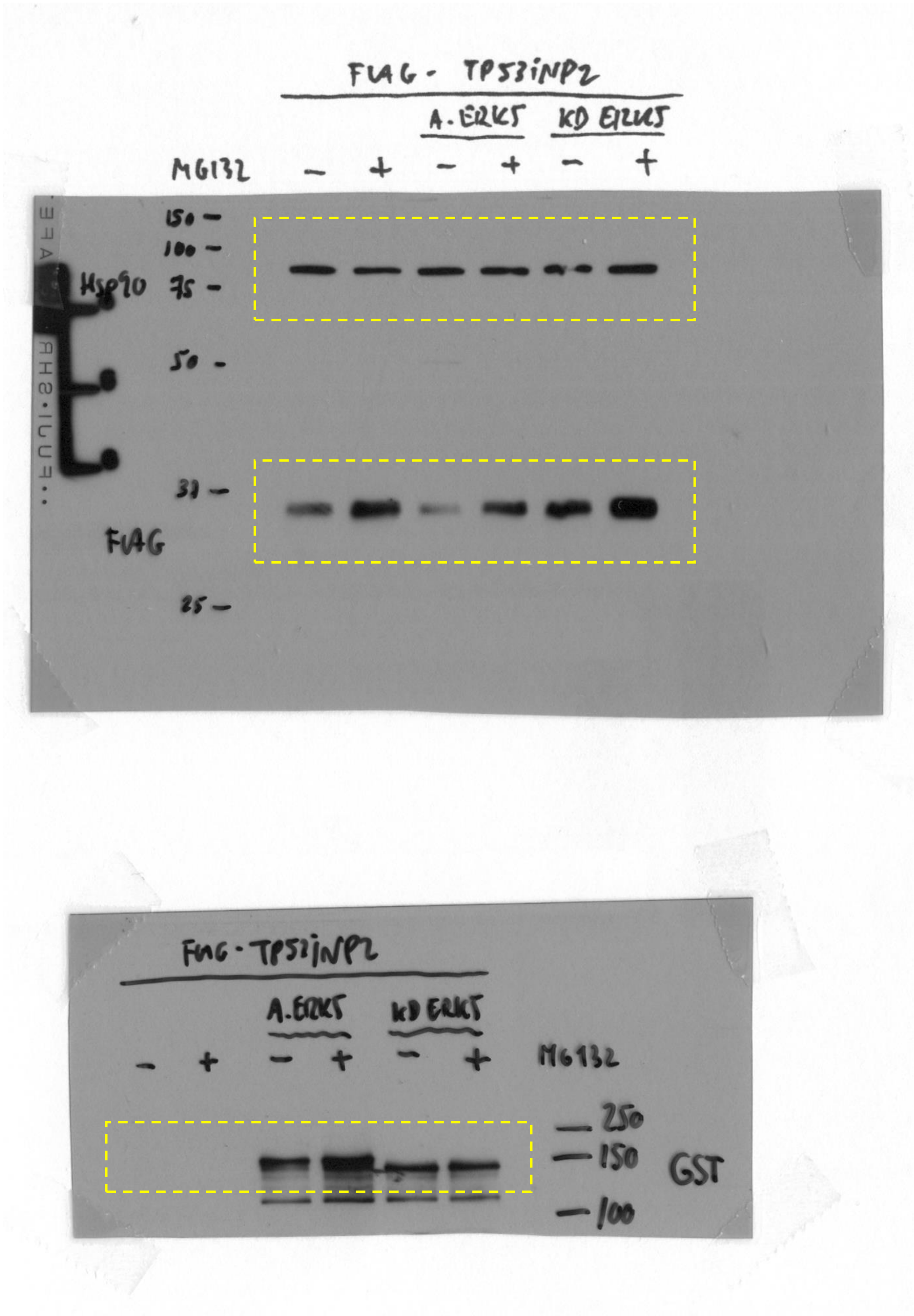

FIGURE 6A AN3CA

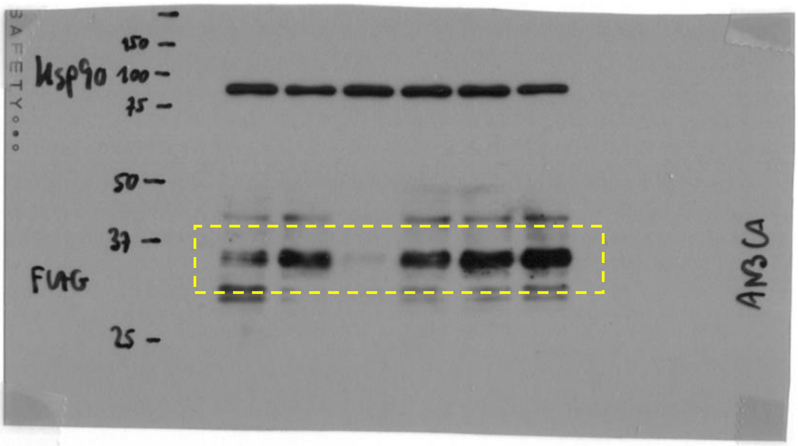

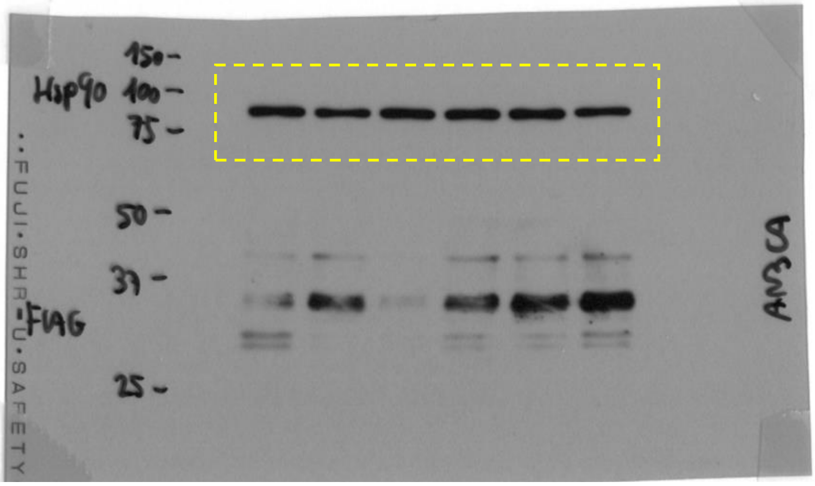

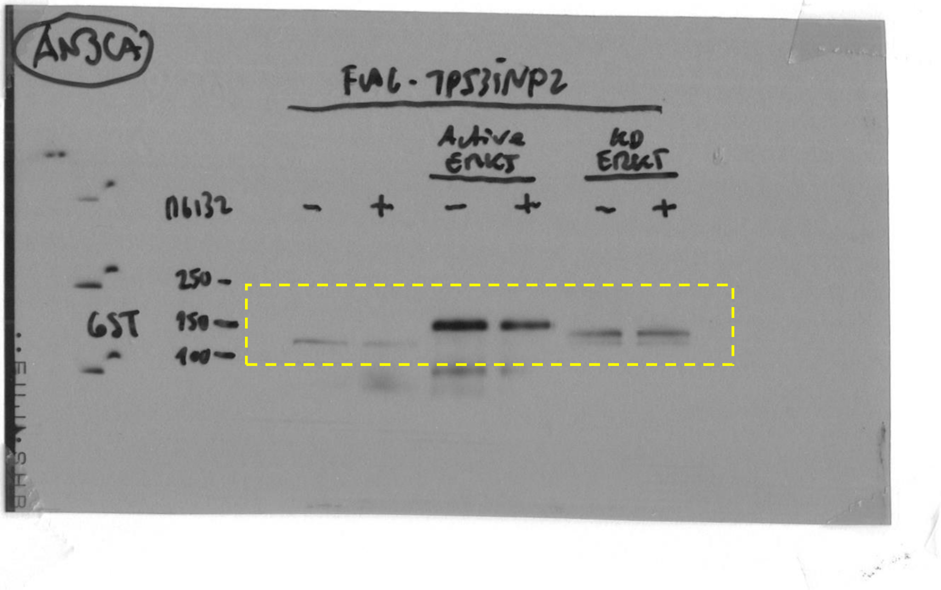

FIGURE 6B

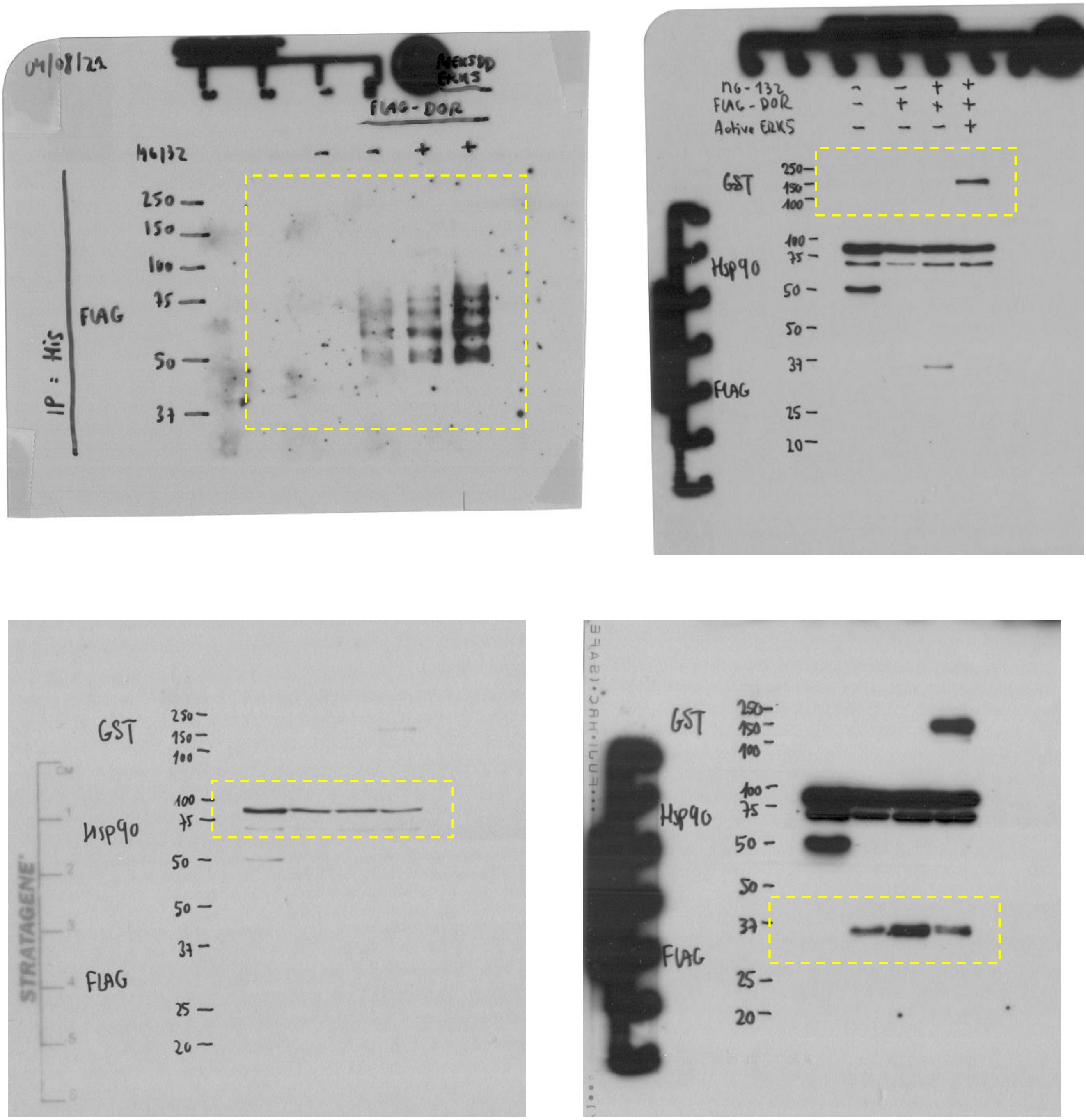

FIGURE 6C Ishikawa and AN3CA cells

Ishikawa

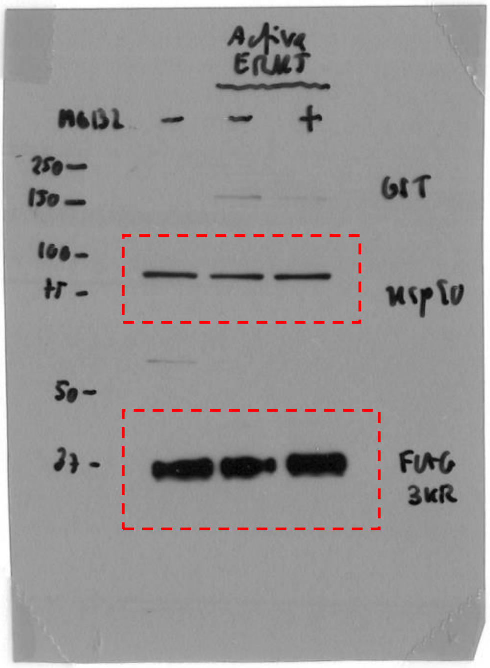

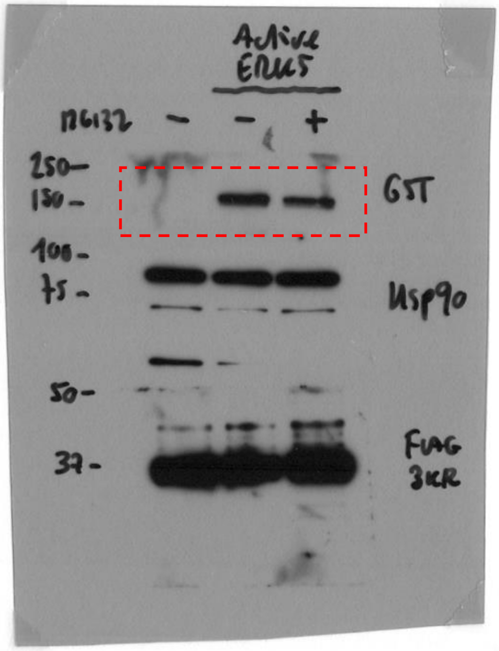

AN3CA cells

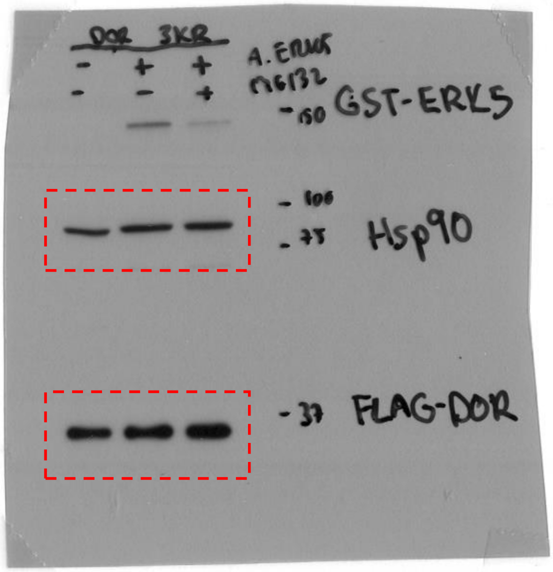

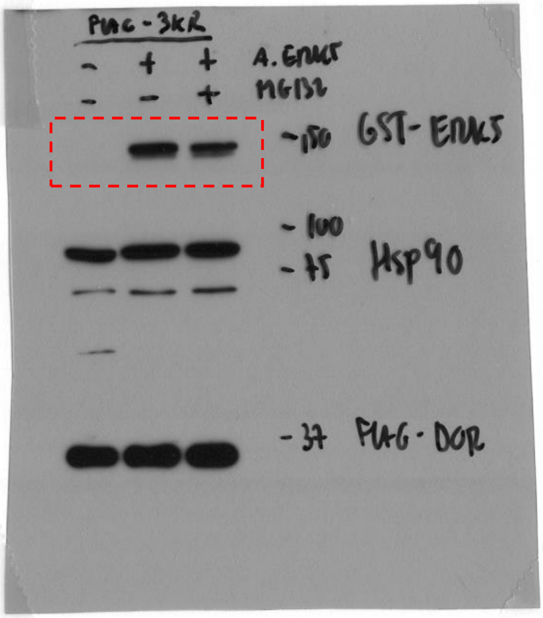

FIGURE 6D Ishikawa

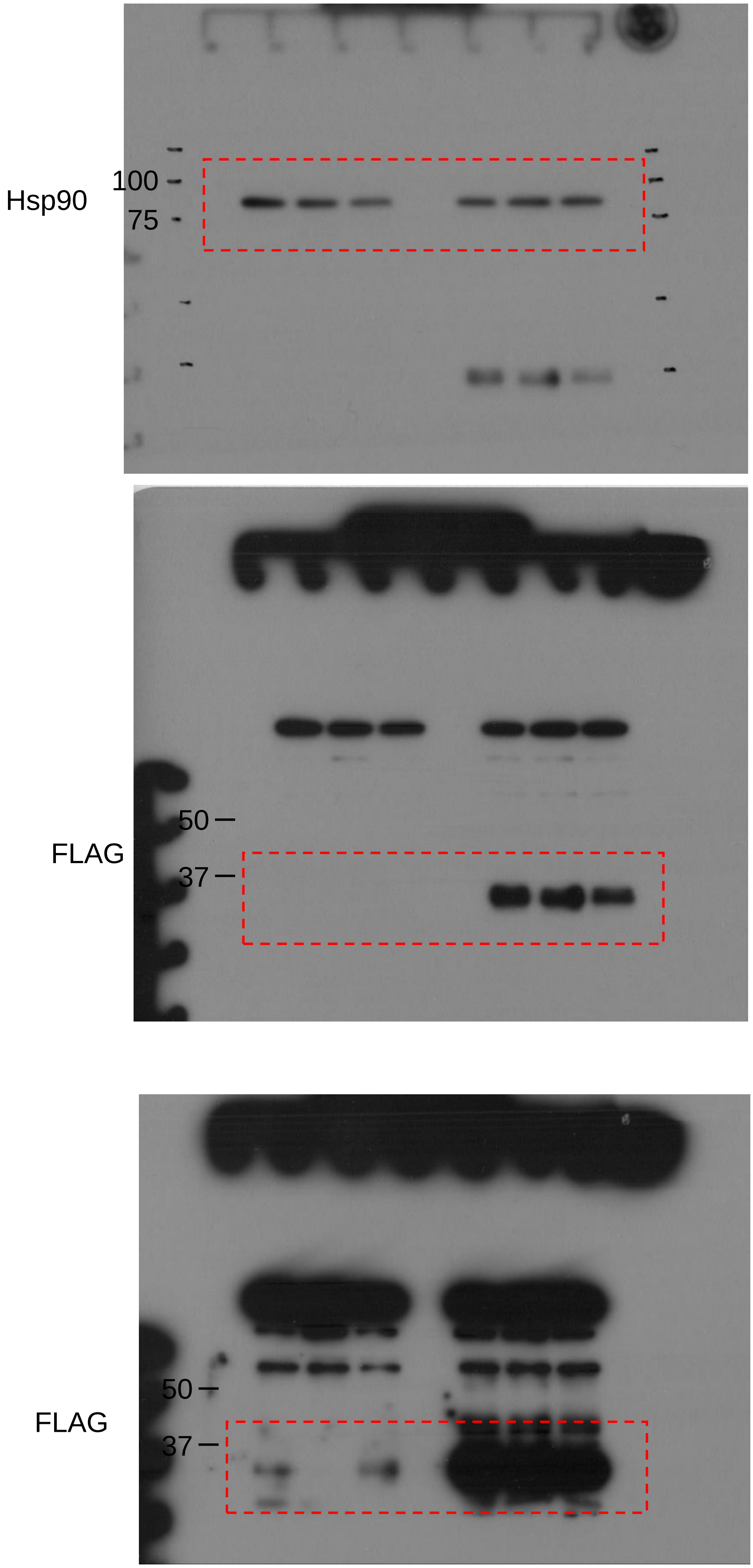

FIGURE 6D AN3CA

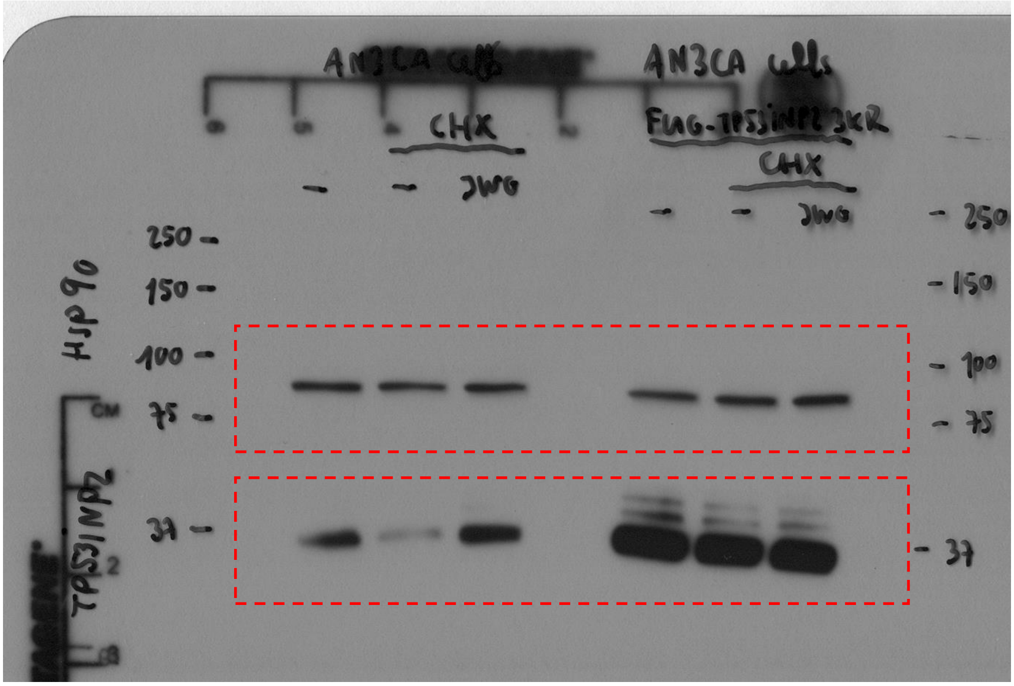

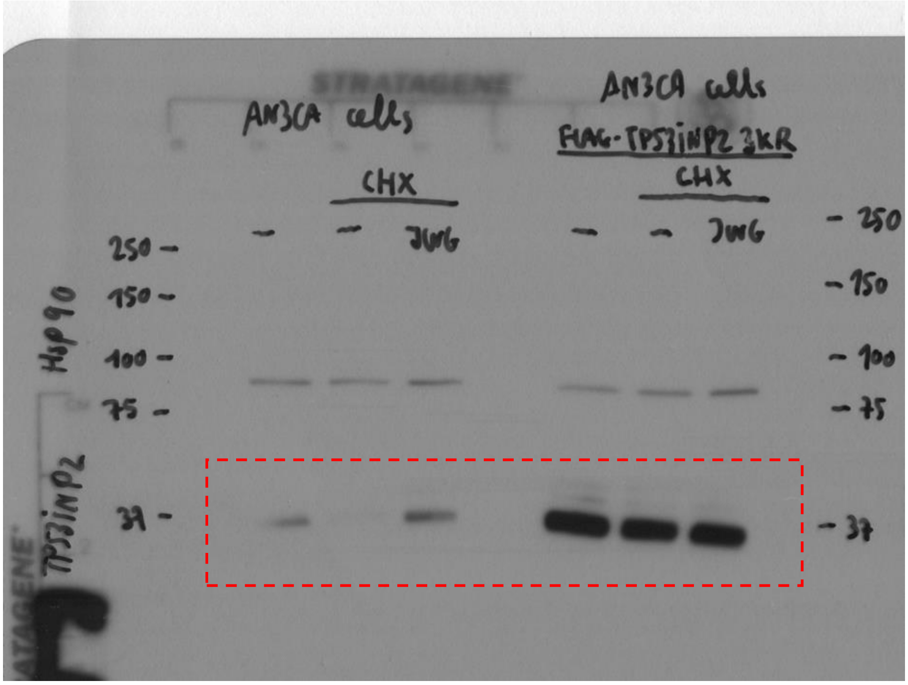

FIGURE 6E

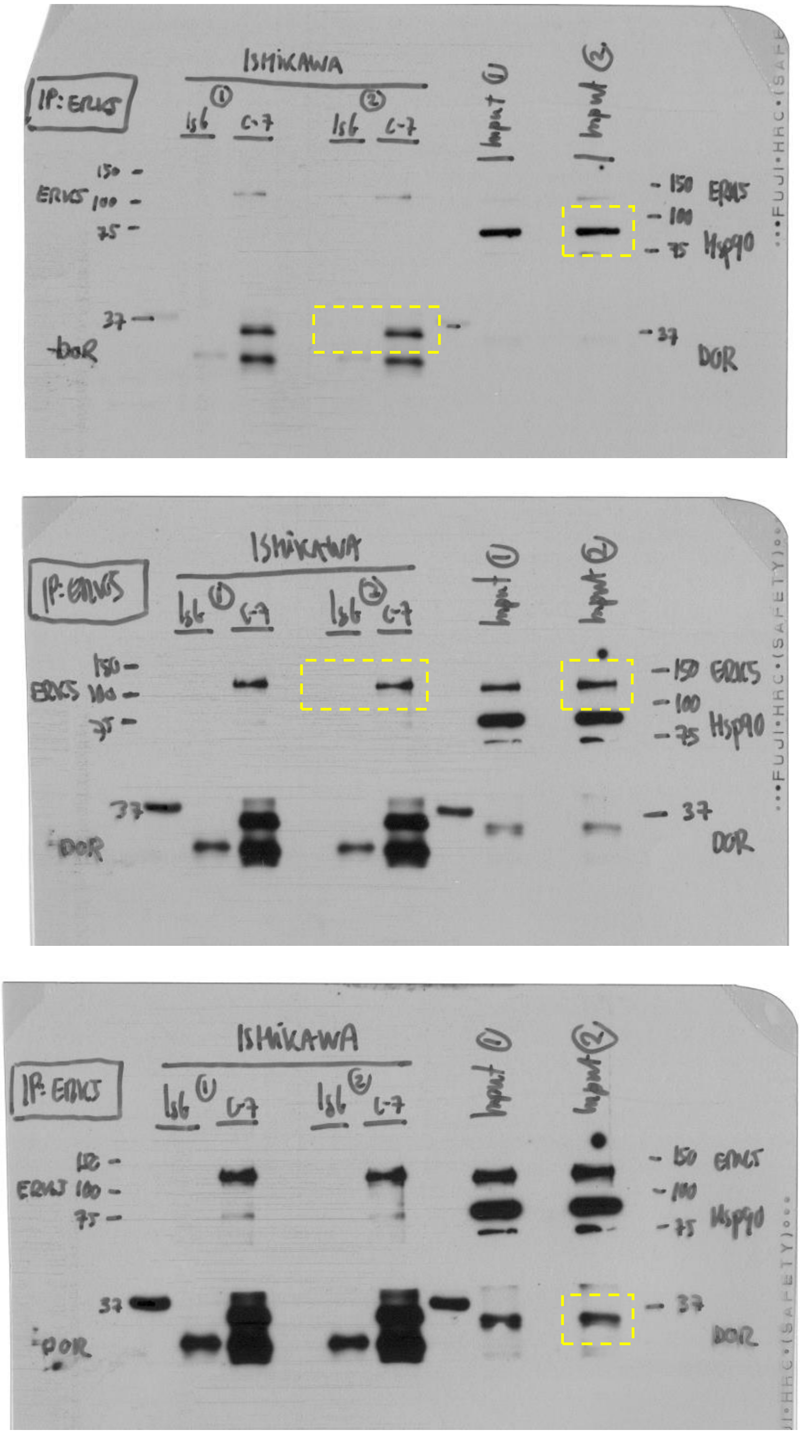

ISHIKAWA
IP: ERK5
IgG
C-7
Input 1
Input 2
150
100
75
37
ERK5
Hsp90
DOR

FIGURE 6F

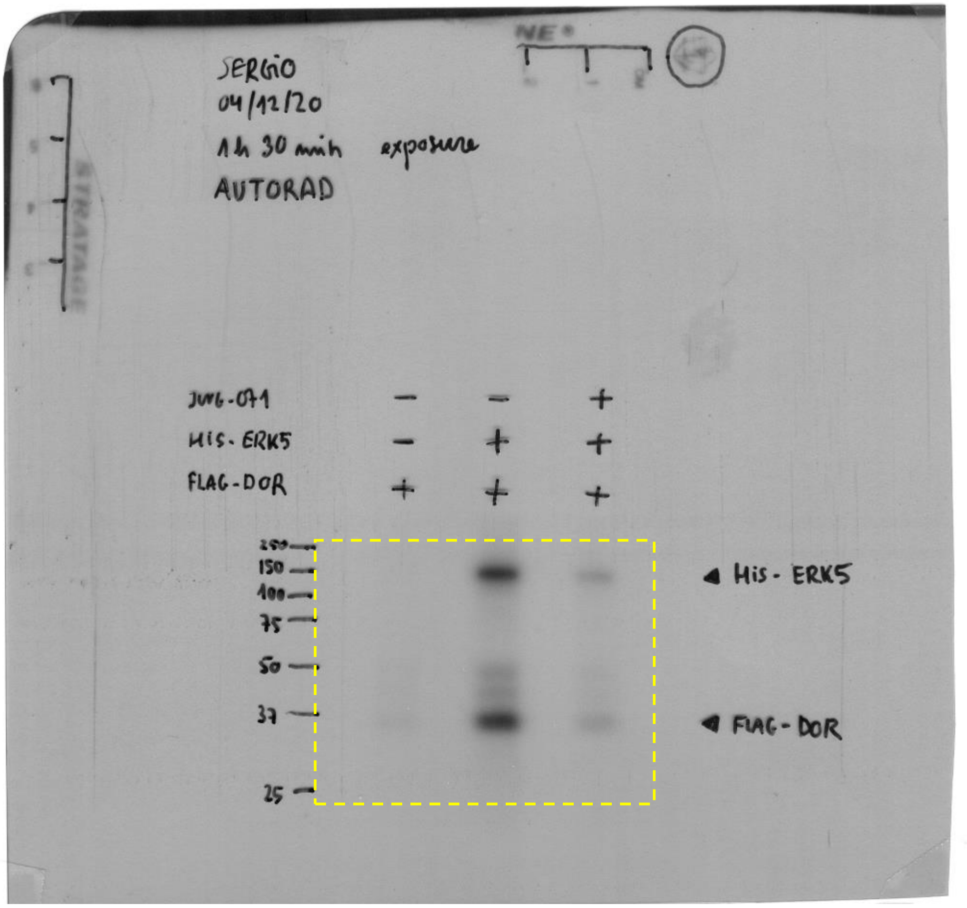
SERGIO
04/12/20
1h 30 min exposure
AUTORAD
JWG-071 − − +
His-ERK5 − + +
FLAG-DOR + + +
250
150
100
75
50
37
25
His-ERK5
FLAG-DOR

FIGURE 6H

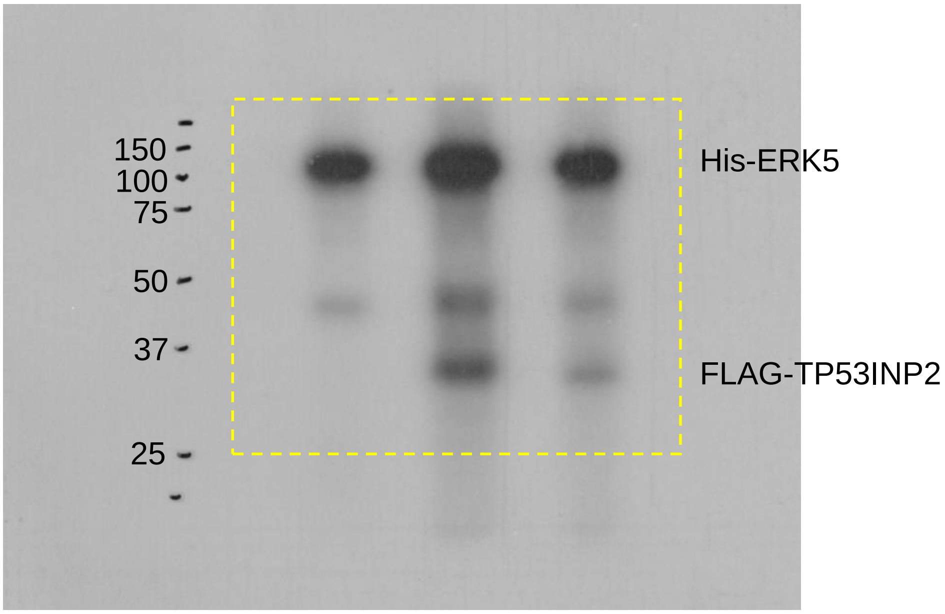

FIGURE 6I Ishikawa

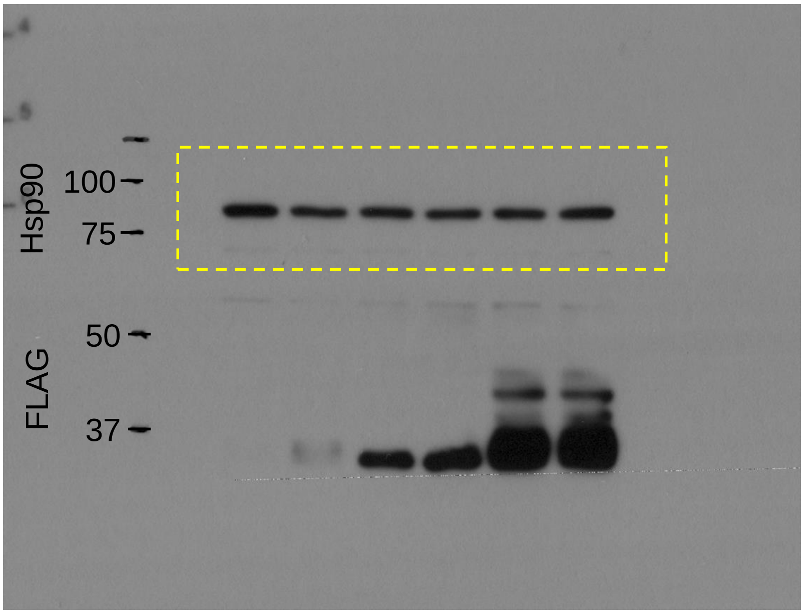

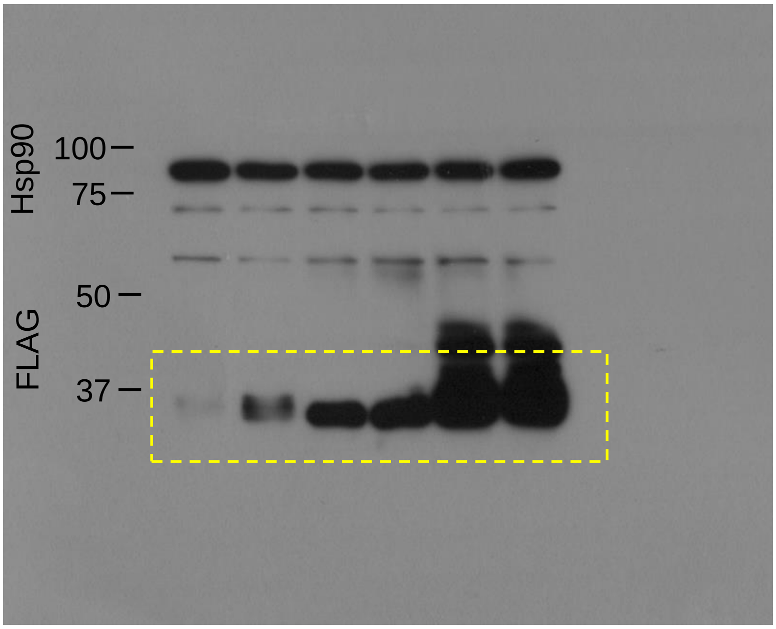

FIGURE 6I AN3CA

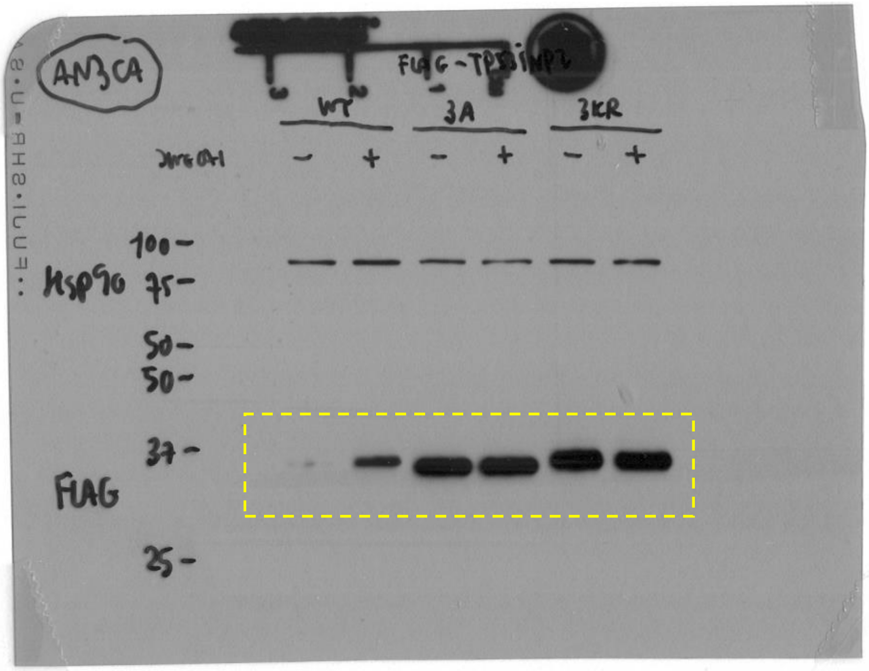

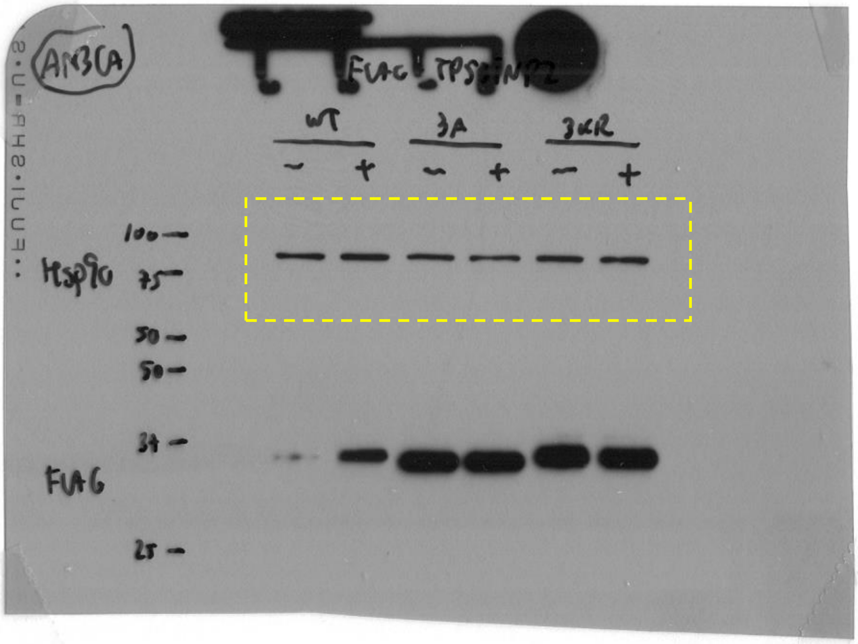

FIGURE 6J

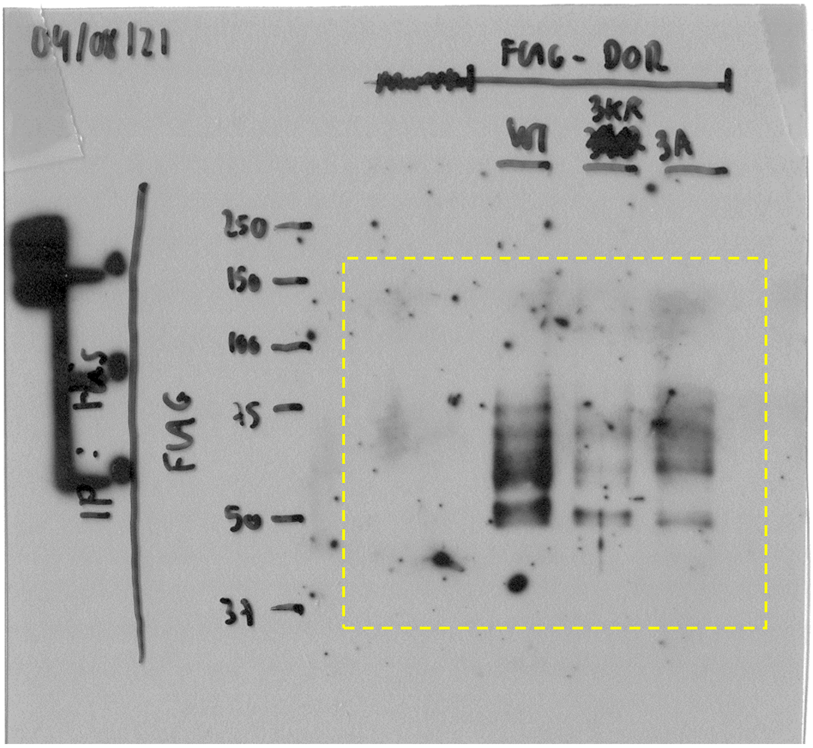
04/08/21
FLAG - DOR
WT
3KR
3A
250
150
100
75
50
37
FLAG
IP : His

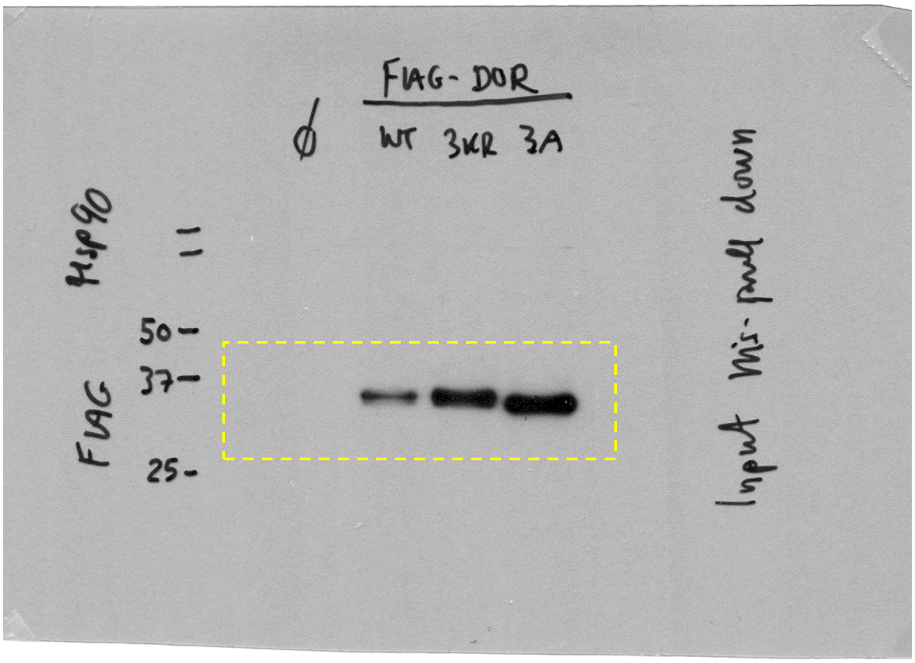
FLAG - DOR
Ø
WT
3KR
3A
Hsp90
50
37
25
FLAG
Input His-pull down

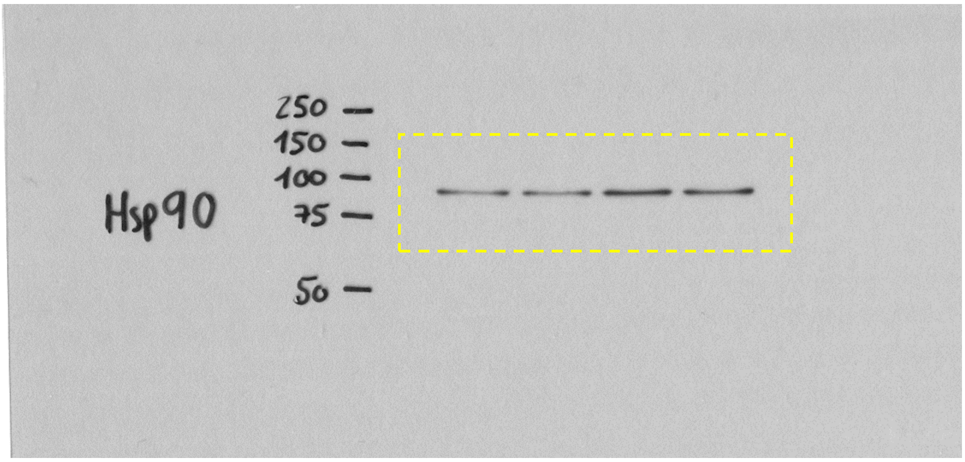
250
150
100
75
50
Hsp90

FIGURE 6K

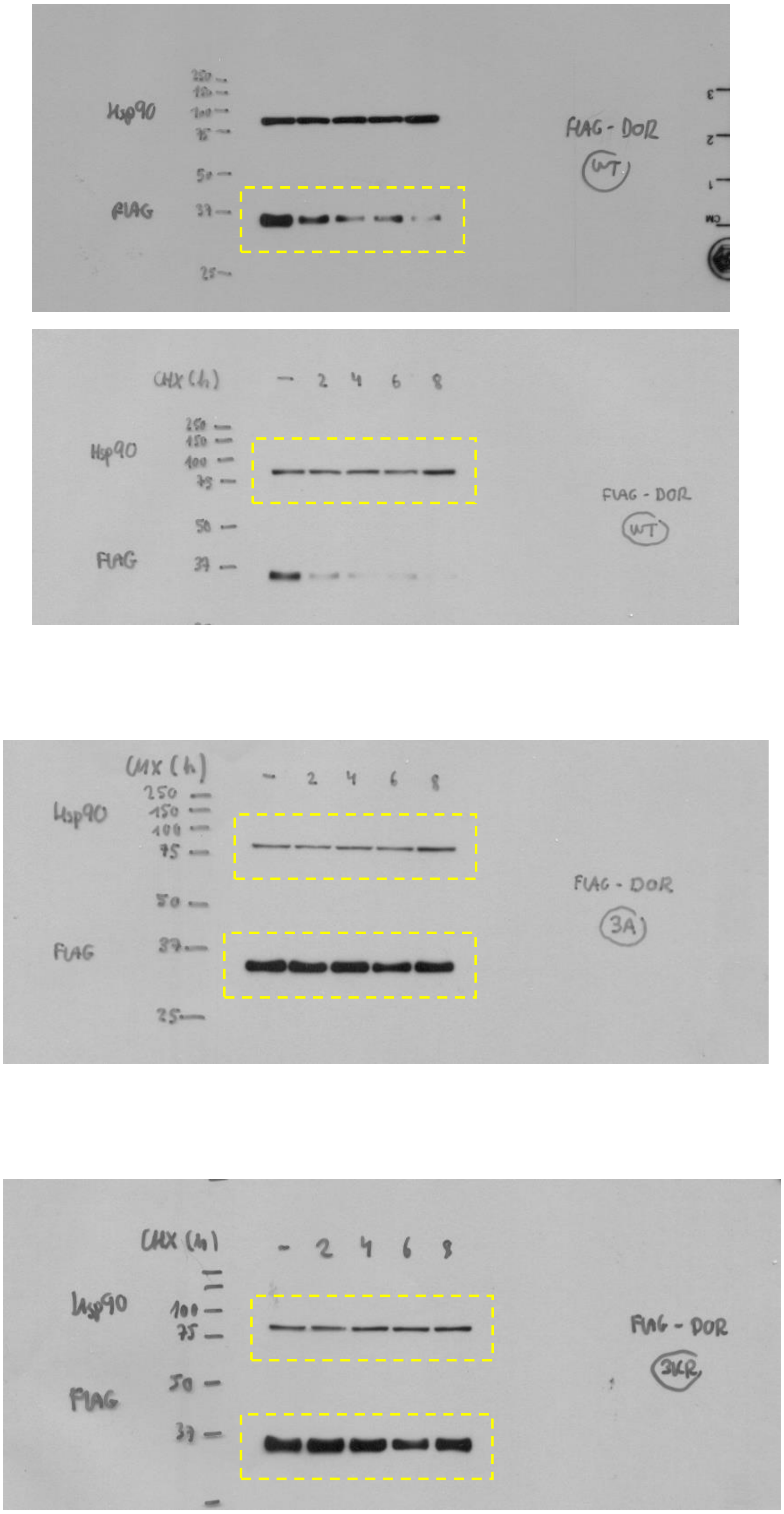
Hsp90
FLAG
FLAG - DOR
WT
CHX (h)
- 2 4 6 8
250
150
100
75
50
37
25
FLAG - DOR
WT
CHX (h)
- 2 4 6 8
Hsp90
FLAG
FLAG - DOR
3A
CHX (h)
- 2 4 6 8
Hsp90
FLAG
FLAG - DOR
3KR
